# Structural Implications of Mutations Conferring Rifampin Resistance in *Mycobacterium leprae*

**DOI:** 10.1038/s41598-018-23423-1

**Published:** 2018-03-22

**Authors:** Sundeep Chaitanya Vedithi, Sony Malhotra, Madhusmita Das, Sheela Daniel, Nanda Kishore, Anuja George, Shantha Arumugam, Lakshmi Rajan, Mannam Ebenezer, David B. Ascher, Eddy Arnold, Tom L. Blundell

**Affiliations:** 1 Department of Biochemistry, University of Cambridge, Tennis Court Rd., Cambridge, CB2 1GA UK; 2 Molecular Biology and Immunology Division, Schieffelin Institute of Health Research & Leprosy Center (SIH R & LC), Karigiri, Vellore, Tamil Nadu 632106 India; 3 Department of Dermatology, Father Muller Medical College & Hospital, Mangalore, Karnataka 575 002 India; 4 Department of Dermatology, Trivandrum Medical College, Trivandrum, Kerala 695011 India; 5 Department of Biochemistry and Molecular Biology, Bio21 Institute, University of Melbourne, Parkville, VIC 3052 Australia; 6 Center for Advanced Biotechnology and Medicine (CABM), and Rutgers University Department of Chemistry and Chemical Biology, 679 Hoes Lane, Piscataway, NJ 08854 USA

## Abstract

The *rpoB* gene encodes the β subunit of RNA polymerase holoenzyme in *Mycobacterium leprae (M*. *leprae)*. Missense mutations in the *rpoB* gene were identified as etiological factors for rifampin resistance in leprosy. In the present study, we identified mutations corresponding to rifampin resistance in relapsed leprosy cases from three hospitals in southern India which treat leprosy patients. DNA was extracted from skin biopsies of 35 relapse/multidrug therapy non-respondent leprosy cases, and PCR was performed to amplify the 276 bp rifampin resistance-determining region of the *rpoB* gene. PCR products were sequenced, and mutations were identified in four out of the 35 cases at codon positions D441Y, D441V, S437L and H476R. The structural and functional effects of these mutations were assessed in the context of three-dimensional comparative models of wild-type and mutant *M*. *leprae* RNA polymerase holoenzyme (RNAP), based on the recently solved crystal structures of RNAP of *Mycobacterium tuberculosis,* containing a synthetic nucleic acid scaffold and rifampin. The resistance mutations were observed to alter the hydrogen-bonding and hydrophobic interactions of rifampin and the 5′ ribonucleotide of the growing RNA transcript. This study demonstrates that rifampin-resistant strains of *M*. *leprae* among leprosy patients in southern India are likely to arise from mutations that affect the drug-binding site and stability of RNAP.

## Introduction

Leprosy, a chronic infectious disease, is caused by an obligate intracellular pathogen - *Mycobacterium leprae* (*M*. *leprae*), which shares extensive similarity with *Mycobacterium tuberculosis* (*M*. *tuberculosis*) in proteomic and genomic composition^1^. The prevalence rate of the disease has gradually declined from 21.1 cases per 10,000 people in 1983 to 0.2 cases per 10,000 people in 2015, and this was achieved with the use of the World Health Organization (WHO) regimen of Multidrug Therapy (MDT)^2^. The annual new case detection rates (ANCDR) were reduced from 5.2 million in 1983 to ~287,000 in 2005. Over the past decade, the ANCDR has remained stable, with 214,783 new leprosy cases reported globally in 2016, of which 135,485 were reported from India alone^2^. Development of drug resistance for anti-leprosy drugs was first declared for dapsone in 1964, rifampin in 1976 and ofloxacin in 1996^3^. The emergence of drug resistance poses a much greater threat of resurgence due to the lack of effective alternative treatments such as vaccines, combined with poor understanding of the patterns of transmission^4,5^.

The conventional gold standard for detection of drug resistance in leprosy uses mouse footpad propagation (MFP), a slow and technically challenging approach of limited use, with many labs relying only on clinical and molecular methods^6^. The presence of point mutations within genes encoding known drug targets, such as DNA gyrase, the β-subunit of RNA polymerase (RNAP) and dihydropteroate synthases, is widely considered as an important molecular signature for drug resistance in leprosy^7^. However, this can lead to many false positives, and cannot help in determining the magnitude of resistance. It is therefore important to correlate these methods of detection with clinical outcomes in order to develop confidence in identifying drug resistance in leprosy.

Rifampin (also known as rifampicin) belongs to the rifamycin class of bactericidal drugs that attenuate bacterial transcription, especially of *Mycobacteriacea*, by inhibiting the enzymatic activity of the β-subunit of RNAP holoenzyme (containing the subunits (ββ′α′α″ωσ)) through steric occlusion of the 5′-ribonucleotide of the growing RNA chain^8^. Missense mutations in the *rpoB* gene are known to induce changes in amino acids that line the rifampin-binding pocket leading to structural changes and phenotypic resistance to rifampin in *M*. *leprae*^9^. Mutations usually occur between codon positions 410–480, known as the rifampin-resistance-determining region (RRDR)^10^. There are approximately 40 mutations within the RRDR that possess moderate-to-strong correlation with clinical non-responsiveness to rifampin in MDT^11^ and have been proven using the MFP test and surrogate genetic experiments. Currently, drug-resistance screening is performed only on cases that do not respond clinically to MDT and/or are cases of leprosy relapse. Diagnosis is based on the presence or absence of mutations and is coupled with clinical evidences^12^. Hence identifying mutations within the *rpoB* gene support determination of resistance, which is otherwise dependent to a large extent on the clinical profile of the relapsed leprosy patients. In the absence of an axenic culture medium and where MFP experiments are challenging, it is important to establish a system that enables clinicians and researchers to understand reliably the impact of these point mutations on structural changes, drug binding and loss/gain in function of the drug target. Harnessing computational resources to understand the impact of point mutations on drug-target interactions has emerged as a promising alternative to *in-vitro* experiments and demonstrated substantial agreement with the available experimental evidence^13–21^. Previous studies aimed at understanding the structural consequences of point mutations in the *rpoB* gene of *M*. *leprae* were limited to models based on distantly related homologues used as templates to construct them^22,23^.

In the current study, we have identified point mutations within the RRDR of *rpoB* in clinical isolates from relapsed leprosy cases and non/poor respondents to MDT at three hospitals, which are treating leprosy patients in southern India. To perform more accurate analysis of effects of these mutations on structure and function of RNAP of *M*. *leprae*, comparative models of RNAP were built and structural insights obtained from a recently published crystal structure of RNAP from *M*. *tuberculosis*^8^, which was solved at a resolution of 3.8 Å and shares 96% sequence identity with that of *M*. *leprae*. This structure revealed that rifampin binds to the β subunit of RNAP close to the 5′-end of the growing RNA transcript, where it induces a steric clash with the 5′-ribonucleotide when the transcript is extended by three nucleotides, rendering this ribonucleotide unpaired, unstacked and rotated by approximately ~40°. This clash destabilizes the growing chain and halts transcription. The molecular structures of RNAP of *M*. *tuberculosis* were solved in complex with a synthetic nucleic acid scaffold, which contains a non-template DNA strand passing through a cleft into the active center and with a three-ribonucleotide RNA chain complementary to the template DNA strand. The nucleic acid scaffold is characterized by sequence specific elements that are necessary for recognition by mycobacterial RNAP. However, no mutant structures have been crystallized to date to decipher the structural changes induced by substitution mutations on the rifampin binding site.

We identified the occurrence of rifampicin resistance in leprosy from three different hospitals in southern India. Comparative three-dimensional models of wild-type and mutant RNAP of *M*. *leprae* were generated to evaluate the structural changes that occur due to point mutations and their impact on rifampin binding and stability of transcription in *M*. *leprae*.

## Results

### Clinical findings on follow-up

Clinical and demographic characteristics of the leprosy cases enrolled in the study, were mentioned in Table 1. On monitoring, it was noted that all the cases responded to a second complete round of WHO MDT that was administered for 12 months after the diagnosis of relapse. This response was determined by the absence of new lesions and additional nerve function impairment. One of the possible reasons for this response could be due to the compensating bactericidal and/or bacteriostatic effects rendered by the other drugs in the MDT, the dapsone and clofazimine. We have previously shown that, while *M*. *leprae*-containing rifampin-resistant mutations D441Y and D441V were unaffected by rifampin treatment, administration of the WHO MB regimen of MDT led to reduced bacterial load^24^. It was unclear whether the relapse in leprosy is due to reinfection or presence of persisters in the body from previous infection.

**Table 1:** Clinical and Demographic Characteristics of the Study Subjects.

Si. No.	Characteristics	Types	Relapsed/MDT-Non-Respondent Leprosy Cases	
			Number	%
1	Gender	Male	24	68.57
		Female	11	31.43
2	Age	1–15	0	0.00
		16–30	7	20.00
		31–50	14	40.00
		>50	14	40.00
3	WHO Classification	PB	0	0.00
		MB	35	100.00
4	Ridley Jopling Classification	Indeterminate (IND)	0	0.00
		TT	0	0.00
		BT	5	14.29
		BB	2	5.71
		BL	4	11.43
		LL	24	68.57
5	Current Bacteriological Index (BI)	Negative	3	8.57
		1–2	7	20.00
		2.25–4	14	40.00
		4.25–6	11	31.43
6	MDT Duration	12 Months	18	51.43
		24 Months	10	28.57
		>24 Months	7	20.00
7	Clinical Status	Relapse	13	37.14
		Non-Respondents	22	62.86
8	Duration between initial disease/treatment episode and relapse	0–3 Years	23	65.71
		4–10 Years	7	20.00
		>10 Years	5	14.29

### Mutations detected in *rpoB* gene in relapse/non-respondent leprosy cases

Mutations were detected in four out of the 35 relapse/non-respondent leprosy cases within the rifampin resistance determining region of the *rpoB* gene. The mutations identified were D441Y, D441V, S437L and H476R (Table 2). Sequence chromatograms with mutated regions are shown in Supplementary Material 1.

**Table 2:** Clinical Characteristics of the Cases with Mutations in the *rpoB* Gene.

Si. No.	Lab ID	Age/Sex	WHO/RJ	Previous /Current BI	Previous MDT Details	Time duration before relapse	Classification	Mutation in *rpoB*
1	DRS009	38/M	MB/LL-Histoid	3.5+/3+	2 Years MB MDT	8 Years	Relapse	*H476R*
2	DRS019	45/F	MB/LL	NA/5+	2 Years MB MDT	10 Years	Relapse	*S437L*
3	DRS027	58/M	MB/LL	5+/4+	24 Pulses of MB MDT	6 Years	Relapse	*D441Y*
4	DRS029	62/M	MB/LL	6+/3.66+	2 Years MB MDT	5 Years	Relapse	*D441V*

### Rifampin binding in *M*. *leprae* RNAP

**Figure 1:**
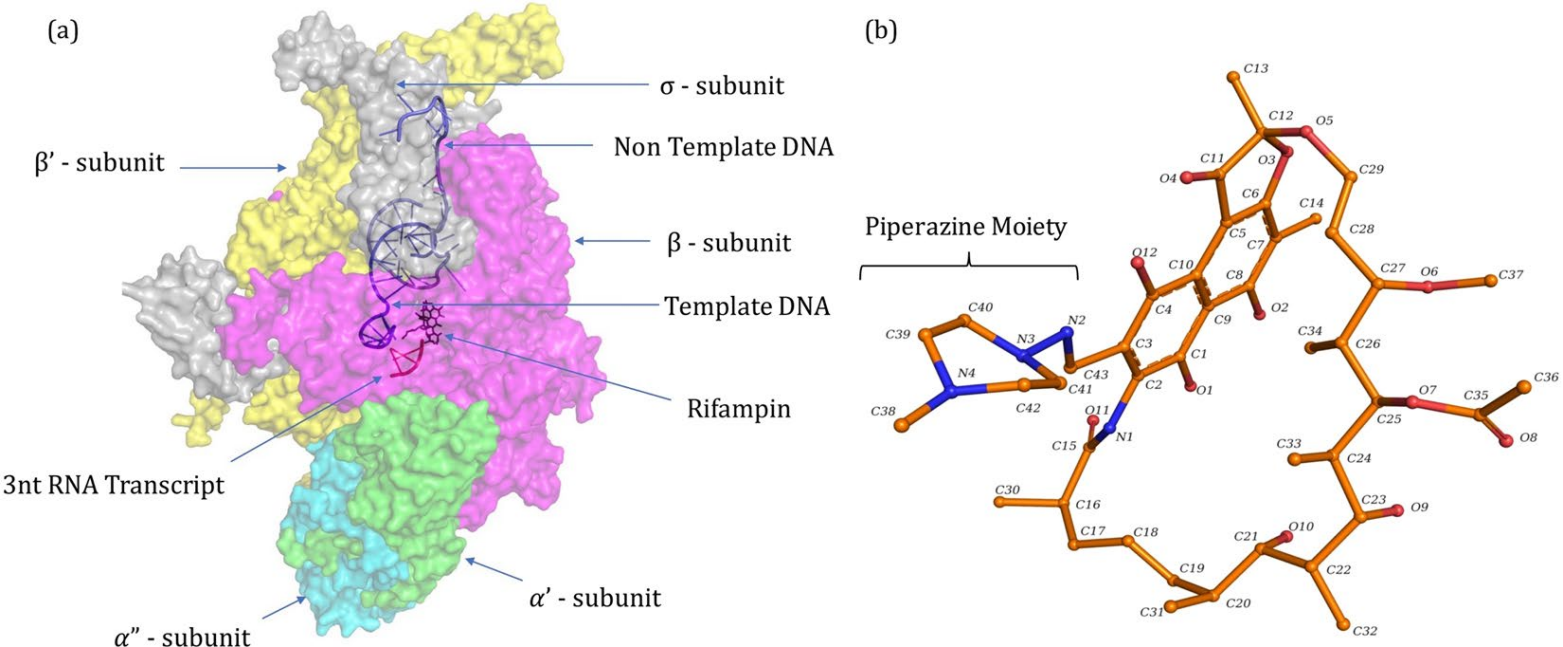
[**a**] RNAP holoenzyme of *M*. *leprae* in complex with synthetic nucleic acid scaffold and rifampin. [**b**] Heterocyclic structure of rifampin (orange) with the piperazine moiety and naphthoquinone core that is spanned by an aliphatic chain.

**Figure 2:**
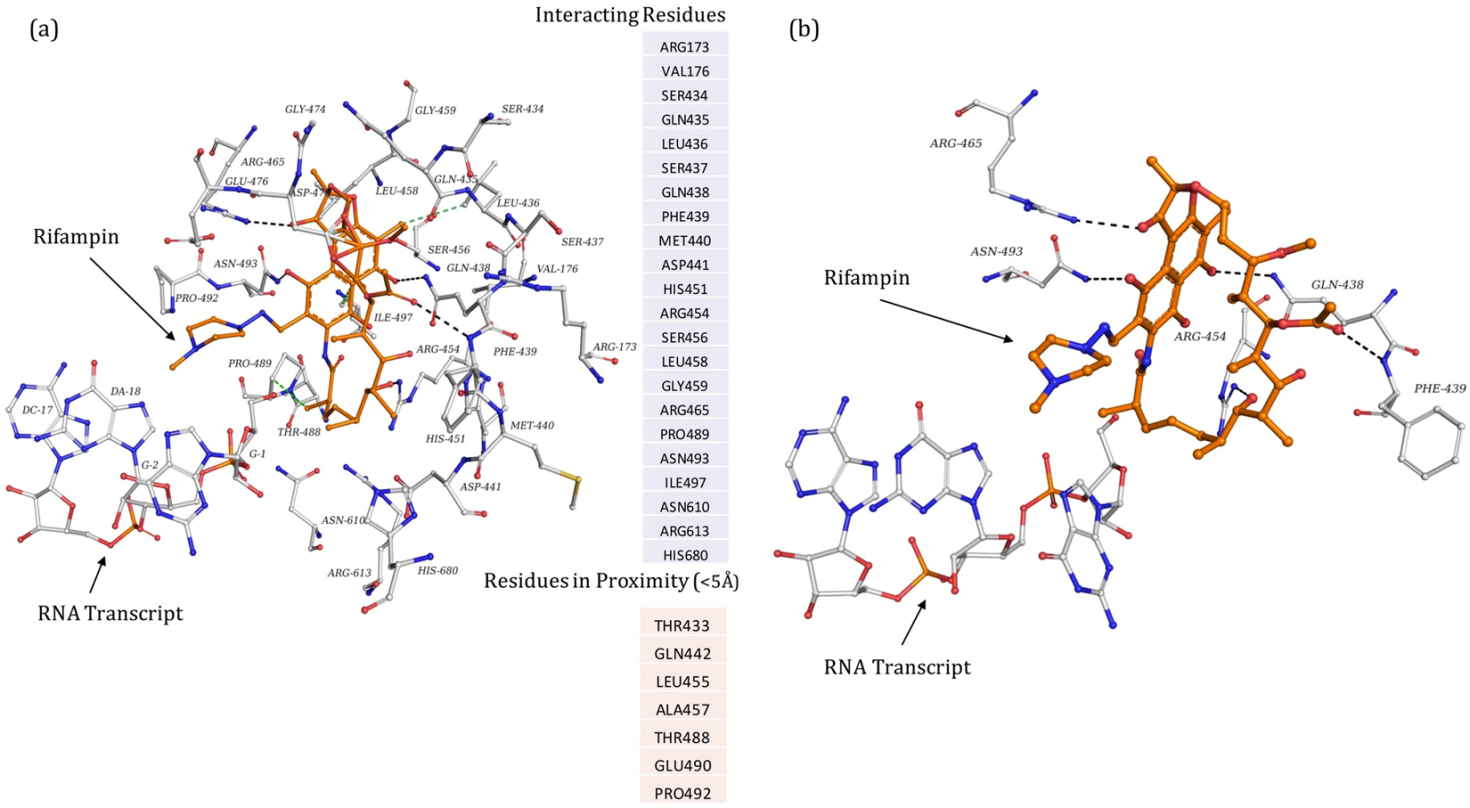
[**a**] Interatomic interactions of rifampin (orange) with the β subunit of RNAP. All the interacting and proximal residues were depicted. The black dashed lines indicate hydrogen bonding interactions and green dashed lines indicate hydrophobic interactions. [**b**] Hydrogen bonds (black dashed lines) between rifampin and sidechains of R454, R465 and N493, and the mainchain of Q438 and F439.

The surface representation of RNAP holoenzyme model of *M*. *leprae* along with the nucleic acid scaffold and molecular structure of rifampin were depicted in Fig. 1. The model shows that rifampin binds to the β subunit of RNAP (Chain C) by forming five hydrogen bonds with the sidechains of R454, R465 and N493, and the mainchain of Q438 and F439 (Fig. 2). Additionally, it also forms weak hydrogen, hydrophobic and π interactions with the surrounding residue environment. The piperazine moiety of rifampin^25^ induces a steric clash with the base of the 5′-ribonucleotide, resulting in unstacking and loss of base pairing with the RNA transcript after it is extended to three nucleotides^8^. As the activity of rifampin relies on its ability to induce a steric clash with the 5′-ribonucleotide, mutations that influence its orientation in the binding pocket might lead to reduction in these steric clashes resulting inseset_name sele rifampin resistance.

### Rifampin binding in mutant *M*. *leprae* RNAP

#### D441Y and D441V

**Figure 3:**
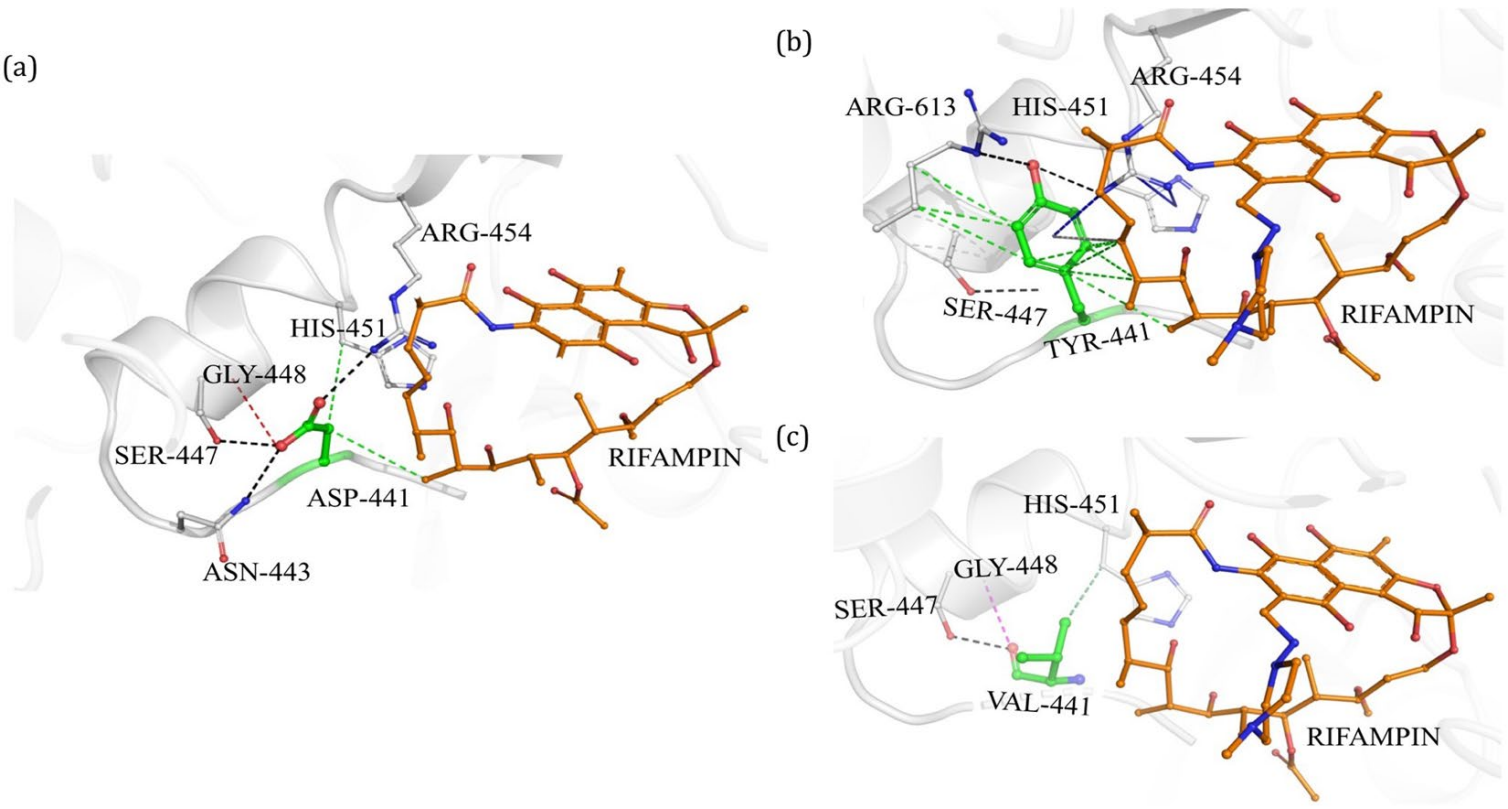
[**a**] Interatomic interactions of D441 (green) with the residue environment in wild-type complex. D441, located at a distance of 3.43 Å from rifampin (orange), makes hydrogen bonds with side chains of S447, R454 and N443 (black dashed lines), a carbonyl interaction with G448 (red dashed line) and proximal hydrophobic interactions with rifampin, and H451 (green dashed lines). [**b**] Mutant Y441 interactions with the residue environment. Y441 makes hydrogen bonds (black dashed lines), hydrophobic interactions (green dashed lines), carbon-π interactions (grey dashed lines) and donor-π interactions (blue dashed lines). [**c**] Mutant V441 interactions with the residue environment. Hydrogen bonding, hydrophobic and carbonyl interactions are depicted.

Aspartate at the position 441 in the wild-type complex (Fig. 3a) is located within the rifampin binding pocket and forms several interactions with the surrounding residues, some of which include backbone and sidechain hydrogen bonds with S447, R454 and N443, a carbonyl interaction with G448 and proximal hydrophobic interactions with rifampin, and H451. The sidechain oxygen atom forms two weak hydrogen interactions with carbons 20 and 21 of the rifampin, which may stabilize the binding of rifampin. Mutation D441Y (Fig. 3b) induces changes in the binding pocket by introducing an aromatic sidechain into the pocket, which destabilizes hydrogen-bonding interactions with rifampin. It forms many hydrophobic interactions with R613 and rifampin. It also forms donor-π and carbon-π interactions with R454 and rifampicin which might impact the shape and conformation of the rifampin binding pocket. The D441V (Fig. 3c) mutation results in the loss of hydrogen bonds with the surrounding residues that may contribute to conformational changes, leading to resistance.

#### S437L

**Figure 4:**
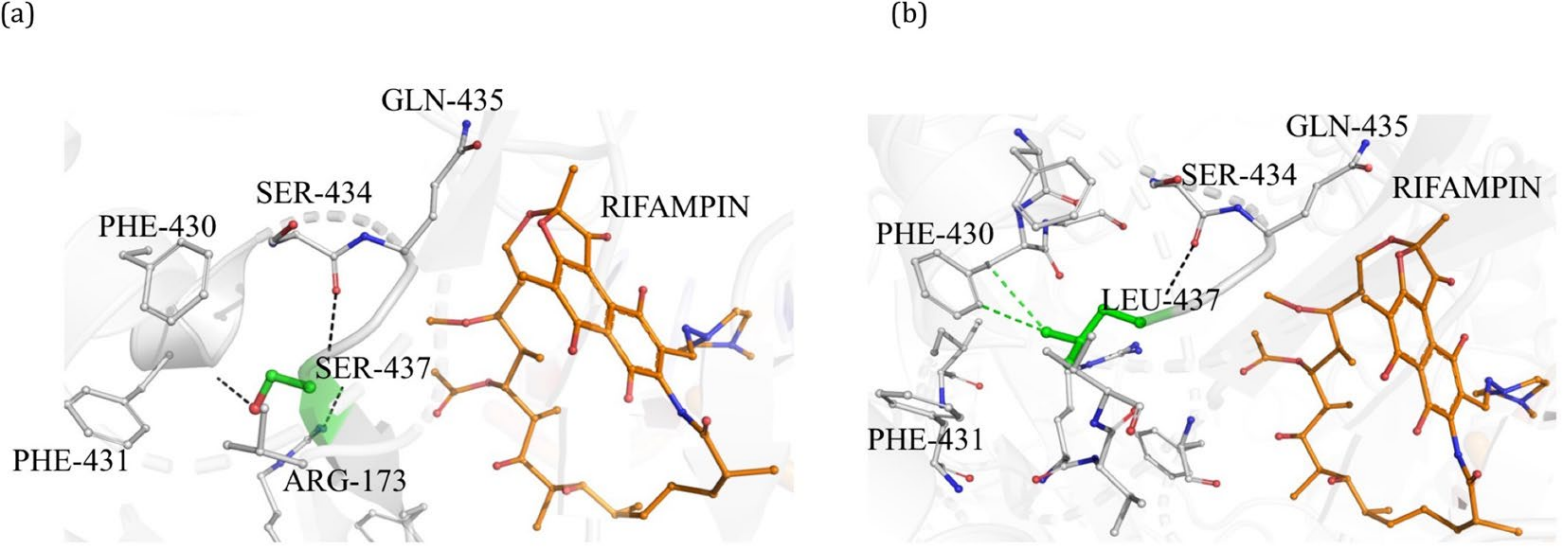
[**a**] Interatomic interactions of S437 (green) with the residue environment in the wild-type complex. Hydrogen bonding interactions with S434, G432 and R173 (black dashed lines) were depicted. [**b**] Mutant L437 with hydrogen bonding and hydrophobic interactions with the surrounding residues.

In the wild-type complex, S437 is located at a distance of 4.03 Å from bound rifampin and is involved in a mainchain hydrogen bonding interactions with S434 and G432, and a sidechain hydrogen bond with R173 (Fig. 4a). The residue is stabilized by various proximal polar interactions, which might contribute to the structure of the rifampin-binding pocket. In the mutant state, S437L (Fig. 4b) forms one mainchain hydrogen bond with S434 and introduces an aliphatic leucine sidechain in the binding pocket, which forms several hydrophobic interactions with surrounding residues. Hence, loss of hydrogen-bond interactions coupled with the development of a hydrophobic environment may impact the orientation of the rifampin in the binding site and lead to resistance.

#### H476R

**Figure 5:**
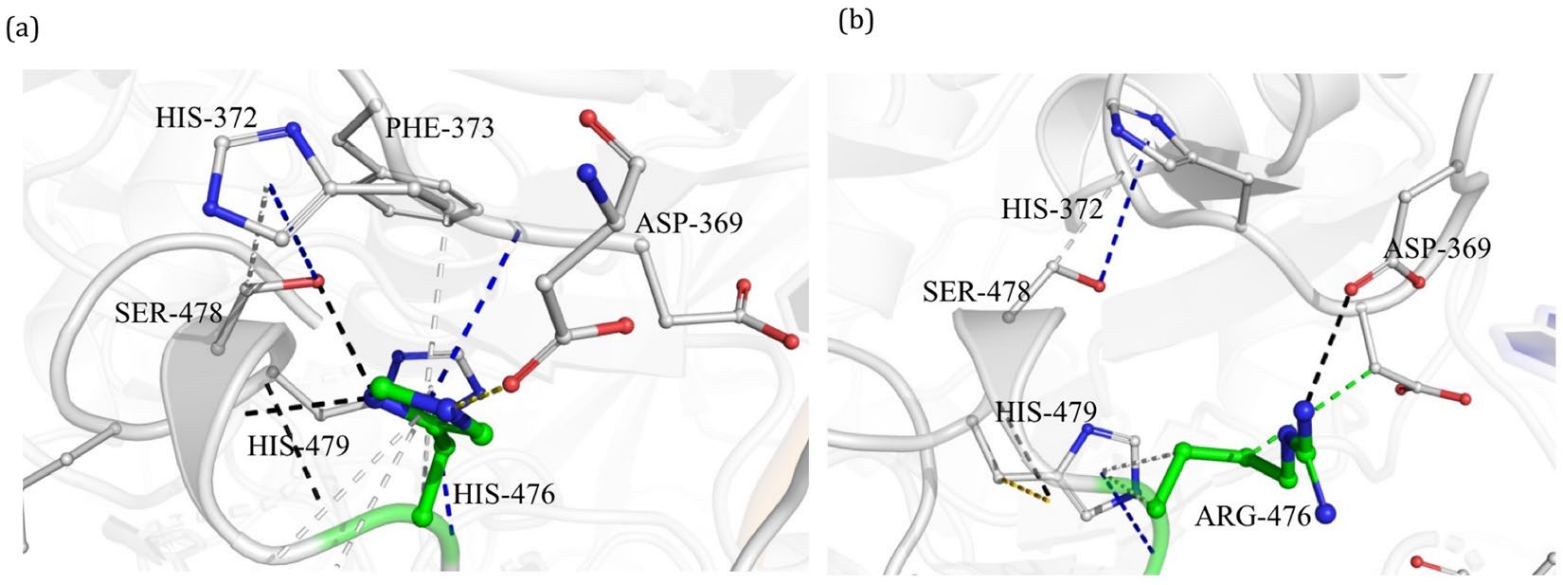
[**a**] Interactions of H476 with the residue environment in the wild-type complex include hydrogen bonds ((black dashed lines), hydrophobic interactions (green dashed lines), ionic interactions (yellow dashed lines), donor-π interactions (blue dashed lines), carbon-π interactions (grey dashed lines). [**b**] Mutant R476 interactions with the residue environment.

H476 is comparatively far from the drug-binding site but is present on the loop that supports the alpha-helix in the binding pocket (Fig. 5a). It is located at 16.74 Å from rifampin and has a dense network of ionic, carbon-π and donor-π interactions that contribute towards stability. The side and mainchains of H476 forms three hydrogen bonding interaction, two with S478 and one with H479. It also forms an ionic interaction with D369. H476 and H372 are involved in edge-to-face ring interactions, with the distance between the centroids being 5.5 Å and angle between the planes is 77.8°. There are also donor-π and carbon-π interactions with the backbone of H372. The mutation H476R would lead to a loss of these ring-ring and ring-atom interactions, which might result in the alpha helix moving towards the binding pocket leading to resistance through an allosteric mechanism (Fig. 5b).

### Conservation of mutating residues in wild-type RNAP of *M*. *leprae*

The wild-type residues D441, S437 and H476 are well conserved across mycobacterial species. Multiple sequence alignment (using MUSCLE-alignment tool^26^) of the β-subunit of RNAP from *M*. *leprae*, *M*. *tuberculosis*, *M*. *abscessus*, *M*. *ulcerans* and *M*. *kansasii* showed that all the above three residues were conserved (Supplementary Material 1). We also used ConSurf server^27^ to understand the degree of evolutionary conservation of each of the residues in a probabilistic framework and noted that the scores for residues in rifampin binding pocket to be less than zero indicating that they are conserved (normalized conservation scores D441 = −0.804, S437 = −0.841 and H476 = −0.805).

### Structural and Functional Consequences of *rpoB* Resistance Mutations

We used three different sets of tools to understand the changes in stability and interactions between subunits, ligands and nucleic acids in the RNAP complex. The impacts of mutations on thermal stability were determined using Site Directed Mutator (SDM)^14^ which considers mutations in specific residues based on their local structural environments where substitution probabilities are calculated from analysis of families of protein homologues. Later we used a machine learning approach called mutation cut-off scanning matrix (mCSM)^13,15^ that uses the pharmacophore properties of the mutating residues and calculates the changes in stability of protein-protein, protein-nucleic acid and protein-ligand interactions. Finally, we used, a vibrational entropy and enthalpy-based approach (ENCoM and FoldX4)^28,29^ to measure the impact of mutations on the flexible conformations of RNAP. Prediction of stability of RNAP complex by mCSM^15^ indicated a stabilizing effect for mutations D441Y and D441V while slightly destabilizing effect for S437L and H476R (Table 3). Both the D441Y and D441V mutations were known to exert strong resistance to rifampin in leprosy as studied by experimental approaches. Enhanced stability of the RNAP complex in the presence of these mutations might sustain bacterial transcription even in the presence of rifampin. This was also supported by the mCSM-lig predictions where all the mutations in the current study were predicted to destabilize the RNAP-rifampin interactions. Additionally, with both the D441Y and D441V mutations, an enhanced stability of protein-nucleic acid interaction was noted indicating that these mutations may sustain protein-DNA and protein–RNA interactions and subsequent transcription (Table 3). Except for H476R, all the other mutations lie within a 4 Å distance from the ligand and in the rifampin binding pocket. With S437L, both the protein as well as the protein- ligand interactions were predicted to be destabilized by mCSM and mCSM-lig, indicating that this mutation not only impacts the binding of rifampin but also alters the function of the β subunit of RNAP in transcription. Although located away from the rifampin binding site, the H476R mutation was predicted to destabilize the protein and its interactions with ligand, nucleic acids and the interface. It was noted that this residue lies at the interface between the β and β′ subunits of RNAP and mutation may destabilize the interface of the subunits with −1.171 kcal/mol of change in energy (measured using mCSM-ppi). ENCoM and FoldX4 programs predicted destabilizing energy changes in RNAP in the presence of all the four mutations. By generating normal modes using elastic network contact model and calculating the vibrational entropy and enthalpy changes in the system, it was noted that the current mutations impact the thermodynamic properties of RNAP and has potential effects on conformational flexibility.

**Table 3:** Effect of Mutations on Protein Stability and Interactions with Ligand, Nucleic acid and Other Protein Subunits.

Impact of Mutations on Protein Stability and Ligand Interaction (*ΔΔ*G in kcal/mol) *				
Prediction Tools	D441Y	D441V	S437L	H476R
mCSM	0.042	1.513	−0.031	−0.933
mCSM-lig	−0.147	−0.204	−0.268	−0.516
mCSM-NA	5.990	0.345	−3.55	−1.008
mCSM-PPI	−0.294	0.155	−0.555	−1.171
DUET	0.002	1.597	0.190	−0.956
SDM2	−0.710	0.330	1.280	−1.870
ENCoM & FoldX4**	2.020	1.210	1.880	2.110
Interface Residue	No	No	No	Yes
Distance from the Interface	7.080 Å	7.080 Å	7.090 Å	3.550 Å
Distance from the Ligand	3.435 Å	3.435 Å	4.000 Å	16.745 Å
Distance from the Nucleic Acids	7.082 Å	7.082 Å	12.344 Å	8.624 Å

*Except for ENCoM & FoldX4, all the positive values indicate a stabilizing effect while the negative values indicate a destabilizing effect of the mutations.

**The final energy change is calculated as the sum of the predictions by ENCoM and FoldX4 and hence a ΔΔG of >0.5 kcal/mol is considered as a destabilizing mutation.

It was noted that the mutations induced a mild stabilizing effect in the backbone hydrogen bonding (as noted by changes in free energies) when compared to those of sidechain hydrogen bonds, which are destabilizing. As most of the mutated residues lie within the rifampin-binding pocket and their sidechains interact with rifampin, the destabilizing sidechain hydrogen bonding energies positively correlate with the destabilizing effects rendered by mCSM-lig (Table 4). Entropic changes in both sidechains and mainchains of the mutating residues are predicted to have destabilizing effects.

**Table 4:** Energy Changes Calculated by ENCoM and FoldX4 (∆∆G Expressed in kcal/mol).

PDB/Energy Characteristic*	D441Y	D441V	S437L	H476R
Total Energy	2.0281	1.2158	1.8890	2.1128
Backbone Hbond	−0.1296	−0.2289	0.2056	−0.6232
Sidechain Hbond	1.8841	1.7381	0.4473	1.9277
van der Waals	−0.8316	−0.1233	−1.7983	0.2350
Electrostatics	0.4630	0.8407	0.02593	−2.2988
Solvation Polar	−0.6892	−0.8747	0.7040	0.7178
Solvation Hydrophobic	−1.3490	−0.5395	−3.9192	0.7932
van der Waals Clashes	1.6205	−0.3424	5.1669	0.0401
Entropy Sidechain	0.1666	0.0596	0.6761	1.1173
Entropy Mainchain	0.8137	0.7074	0.1137	0.2425
Torsional Clash	0.0549	−0.0349	0.2669	0.0381
Backbone Clash	−0.1666	0.0035	0.3899	0.0677
Helix Dipole	0.0158	0.0028	0.0000	0.0361
Energy Ionisation	0.0091	0.01098	−0.0000	−0.1129

*The final energy change is calculated as the sum of the predictions by ENCoM and FoldX4 and a ∆∆G of >0.5 kcal/mol is considered as a destabilizing mutation.

In the rifampin-bound structure, interactions with rifampin were stabilized by the formation of backbone hydrogen bonds with the residues lining the binding pocket. We noted that hydrogen bonds at position D441 and S437 stabilized the ligand in the binding pocket. Similar observations were made in earlier studies but with models that were built from templates with low identity (~40%)^22,23^. The mutant models indicated a decrease in hydrogen-bond interactions coupled by the loss of other interactions leading to a decline in van der Waals and desolvation energies, and changes in the binding patterns. These observations were noted in earlier studies where an *in-silico* docked model of the RNAP β subunit and rifampin revealed energy changes and variations in hydrogen bonding interactions when mutations were introduced in amino acids that line the drug binding site^8^. Amino acid substitutions not only impacted the hydrogen bonds between rifampin and RNAP but also affected the orientation of rifampin in the pocket which was noted through changes in polar and hydrophobic interactions in mutants.

### Impact of mutations on wild-type and mutant RNAP models in flexible conformations

**Figure 6:**
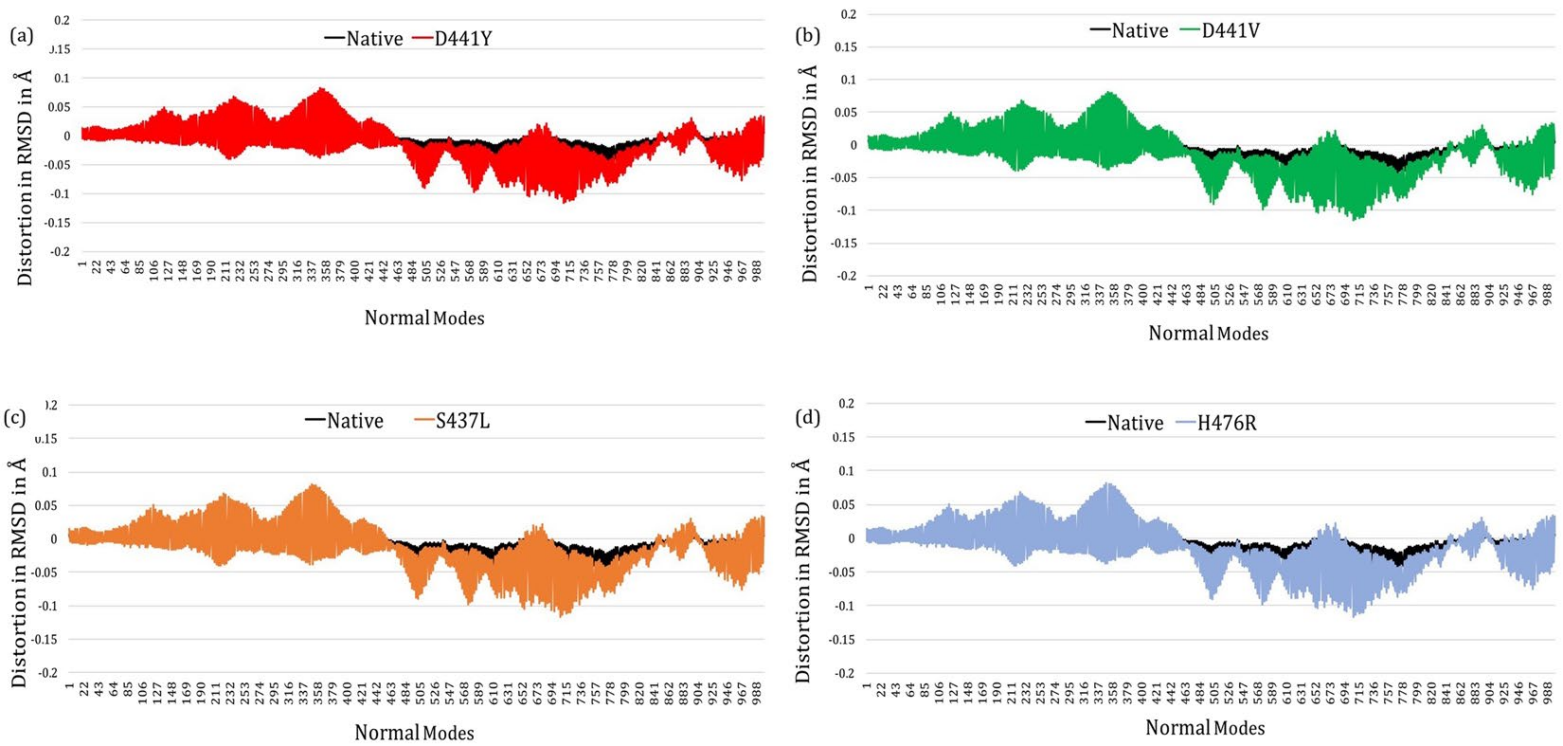
[**a**–**d**] Difference graphs indicating distortion in RMSD (in Å) for each normal mode in the wild-type and in each of the mutant models.

To understand the impact of point mutations on various flexible conformations of the β subunit of RNAP, we applied NMA using ENCoM^28^. NMA was confined to the β subunit of RNAP, and a set of 3387 normal modes were generated for each of the wild-type and mutant models (considering 1129 residues in the β subunit). The distortion in backbone RMSD from the static conformation was noted at each normal mode and compared between wild-type and mutant models. The predicted geometric variations in the C-alpha backbone RMSD under the influence of the first 1000 vibrational normal modes (considering the presence of mutant residues within the first 500 amino acids of the sequence) are shown in Fig. 6a–d. The distortions between the 400–800 normal modes indicate a difference between wild-type and mutant models. This difference in distortion of backbone RMSD of the protein in flexible conformations may impact rifampin binding and the ensuing resistance.

## Discussion

Secondary resistance to rifampin in Indian leprosy patients has been reported from the year 2001^30^. These studies were dependent to a large extent on clinical outcomes and MFP experiments. Leprosy cases whose lesions and BI remain stable without changes during MDT and/or develop new lesions during therapy are clinically suspected as drug resistant. On the other hand, cases that are cured after MDT and develop new signs or symptoms post treatment are categorized as relapse, which can be due to drug resistance or reinfection. A total of 536 relapse leprosy cases were reported in India in 2016^2^; however, only 3–5% of these carry mutations conferring rifampin resistance as noted from the unpublished data collected by WHO Sentinel surveillance study on Drug Resistance in Leprosy^11^. There are recent reports of the emergence of primary resistance to rifampin in leprosy^31^ based on the observation of mutations within RRDR in new cases; however, follow-up is needed to determine the clinical outcomes of resistance as most of these cases may respond to combinatorial therapy (MDT) and are cured.

Four cases that were identified to be drug resistant in the current study, relapsed after an average duration of approximately seven years from the first episode of the disease and complete regimen of MB MDT. It was noted that these cases demonstrate high bacillary load with BI greater than 2+ and belong to lepromatous leprosy. Lepromatous leprosy (LL) is characterized by high bacillary loads and low *M*. *leprae* specific cell-mediated immune responses^32^ in the host, increasing likelihood of the presence of bacterial persisters post-treatment which may adopt to rifampin by inducing point mutations with the RRDR. Most of the rifampicin-resistant strains of *M*. *leprae* that are proven resistant in the MFP approach, harbor mutations in the RRDR of *M*. *leprae* genome^6^. There is no definitive test to differentiate relapse (due to bacterial persisters) from reinfection except for recent observations in whole genome analysis^4^, which is a difficult technique to perform in a diagnostic laboratory setting in tropical countries that are endemic to leprosy. It is therefore not known whether the four cases in the current study were re-infected with the drug-resistant bacilli or possess bacterial persisters that turned resistant to rifampin by inducing mutations over time.

Due to the low level of concordance between MFP, genetic methods and clinical outcomes of drug resistance in leprosy, use of computational methods to understand the structural effects of point mutations provide plausible associations between genetic changes and concurrent phenotypic outcomes. Mechanistic insights into rifampin resistance in leprosy are also important for developing new therapeutic strategies. Surrogate genetic models were used for *M*. *leprae* to determine the magnitude of resistance to rifampin and understand functional outcomes^9^. Mutation D441Y was reported in the year 2001^7^ and was later proved experimentally to cause phenotypic resistance in *M*. *smegmatis*. It was noted that the MIC values have increased by 32-fold in mutant strains indicating that the D441Y mutation can cause strong phenotypic resistance to rifampin^9^. Mutation at codon position 437 was reported by us for the first time in the current study. A very close substitution at the adjacent residue position 436, where leucine is replaced by either proline or serine, was noted in few studies and is known to cause phenotypic resistance to rifampin^33^. Similar mutations were also noted to confer resistance to rifampin in other mycobacteria. The mutation at position 476 conferred rifampin resistance in *M*. *leprae* isolates from Thailand^34^.

Structural characterization of RNAP of *M*. *tuberculosis* revealed that amino acids that are spanning positions 410 to 480 (residues were numbered according to the β subunit of RNAP) line the active catalytic core of the enzyme. The β subunit of RNAP of *M*. *leprae* resembles a “crab-claw” with a cleft that is approximately 30 Å deep. The active site where rifampin binds lie in the central part of the cleft facing the anterior arm. The mCSM-lig predictions support a destabilizing effect on the protein-ligand interactions in all the mutations identified in the current study as they lie within or close to the active site. This program uses an *in-silico* model that relies on interatomic distance graphs, pharmacophore information of the residues and available experimental data on mutations. The predictions made by mCSM-lig correlate with the experimental outcomes of mutations D441Y and D441V. Due to the lack of a quick and robust experimental model to determine the effect of mutations on drug resistance in leprosy, use of predictive tools like mCSM-lig provides important preliminary information on the changes in binding affinity between drug targets and their corresponding ligands.

Wild-type RNAP  in flexible conformation may facilitate proper orientation of rifampin in the active site, enabling the piperazine ring to induce steric clash on the 5′-ribonucleotide of the RNA transcript and repress transcription. Changes in the conformations that are accessible (noted as RMSD distortions at normal modes) in the mutant models suggest that they may impact rifampin binding. This generates structural evidence that these point mutations impact not only the stability but also the dynamics of the RNAP system, leading to phenotypic resistance to rifampin.

The atomic coordinates for RNAP of *M*. *leprae* were predicted from the homologous structure in *M*. *tuberculosis* by comparative modelling considering the spatial restraints. The residues in the rifampin binding pocket of the β subunit between positions 400 and 500 are well conserved across mycobacterial species. All the mutated residues in the current study were conserved between RNAP of *M*. *leprae* and its homologue in *M*. *tuberculosis* as identified from the multiple sequence alignments. Although the model was built from *M*. *tuberculosis* RNAP structure with a resolution of 3.8 Å, it was the best template available with high sequence identity to that of *M*. *leprae*. Available knowledge of the structures of other bacterial RNAPs lends additional credibility as to the reliability of the structure, particularly in the vicinity of the rifampin binding site which is highly conserved across mycobacterial species. The program Andante^35^ considers conserved χ-angle conservation rules from structurally aligned families of homologous proteins when building the mutant models so that the entire side-chain conformations are accurately borrowed, and steric clashes are avoided in the models. The reliability of modest resolution structures is enhanced by the use of the stereochemical restraints in refinement, as is now done routinely and powerfully in modern program suites such as PHENIX^36^.

As *M*. *leprae* is an obligate pathogen and cannot be propagated on an axenic culture medium, it is exceptionally challenging to reconstitute the whole complex of RNAP and ascertain the structural changes experimentally, for each of the mutants. Most of the computational analysis was performed relying on the structural information from RNAP of *M*. *tuberculosis*. While the RNAP model of *M*. *leprae* is highly identical to the experimental structure of *M*. *tuberculosis*, most of the residues in the rifampin binding region are well conserved across mycobacterial species and retain similar interatomic interactions with rifampin. Mutations at residue positions D441 and S437 were noted in rifampin resistant and multidrug resistant strains of *M*. *tuberculosis*^37,38^ and these mutations confer phenotypic resistance to rifampin. Lack of experimental validation of changes in rifampin-RNAP interactions upon mutations becomes a limiting factor for the current study. Although the mutations correlated with the clinical outcomes of rifampin resistance in leprosy (as noted by leprosy relapse), it is important to experimentally validate thermodynamic/structural changes in proteins upon mutations to comprehensively understand the mechanism of drug resistance. The predicted conformational changes provide preliminary insights into the future development of a robust system involving a surrogate model as that of *M*. *smegmatis*, to study the effect of the mutations on MICs^9^ and possibly conformational changes in RNAP if the whole complex can be purified/reconstituted from *M*. *leprae* with nucleic acids and rifampin. A more tractable approach based on currently available Mycobacterial RNAPs that have been crystallized could be to transplant the amino acid residues from the *M*. *leprae* rifampicin-binding region onto either *M*. *tuberculosis* or *M*. *smegmatis* RNAPs and analyse the structural implications of mutations by either crystallography or cryo-electron microscopy.

In conclusion, we have modelled for the first time, a RNAP holoenzyme of *M*. *leprae* using the crystal structure of *M*. *tuberculosis* RNAP as a template. Our experimental results indicate the presence of *rpoB* mutant strains of *M*. *leprae* in southern Indian leprosy patients and *in-silico* modelling provides mechanistic insights into the impacts of these mutations on the binding of rifampin to RNAP in *M*. *leprae*. Three of the mutations that are reported in the current study were identified earlier by other groups and are known to exert strong to moderate resistance to rifampin. The loss of interactions predicted *in-silico* in terms of energy changes and hydrogen bond interactions demonstrate concordance with the *in vitro* experiments that indicated resistance. Similar observations were noted in one of our studies on dapsone resistance in leprosy^39^. Hence this computational approach can further be used to study novel mutations to procure preliminary information on their impacts on structure and function of drug targets and the resulting phenotypic drug resistance in leprosy.

## Methods

### Study Sample

A total of 35 multi-bacillary (MB) leprosy cases, which were clinically diagnosed as relapsed or non/poor respondents to MDT, were enrolled in the study. These patients were diagnosed at the dermatology out-patient department of the “Schieffelin Institute of Health-Research & Leprosy Centre (SIH-R&LC)” in Karigiri–Tamil Nadu, “Father Muller Foundation Medical College–Mangalore, Karnataka and “Government Medical College–Trivandrum in Kerala, in India. A part of the excisional skin biopsy from the site of active skin lesion collected for routine histopathological examination, was sent to the Molecular Biology Laboratory at SIH-R&LC Karigiri for PCR and DNA sequencing to screen for mutations. All the procedures were conducted following the guidelines of the ethical committee of SIH-R&LC–Karigiri - Tamil Nadu, India, and ethical standards as laid down in the 1964 Declaration of Helsinki and its later amendments or comparable ethical standards. Informed and written consent for participation was obtained from all the participants involved the study following the ethical guidelines of SIH-R&LC–Karigiri–Tamil Nadu, India before enrolling in the study. All the experiments conducted in the study were approved by the institutional ethical committee of SIH-R&LC, Karigiri. Demographic and clinical characteristics of the sample are represented in Table 1. According to the WHO, relapse in leprosy is defined as “the multiplication of *M*. *leprae*, suspected by the marked increase (at least 2+ over the previous value) in the bacteriological index (BI) at any single site, usually with evidence of clinical deterioration (new skin patches or nodules and/or new nerve damage)”^40^.

### DNA Extraction

DNA was extracted from skin biopsies using the previously described lysis protocol^41^. Briefly, 25 mg of tissue was manually homogenized, mixed with 1 ml of 1X phosphate-buffered saline (PBS), centrifuged at 8600g for 10 min at 4 °C and the pellet was dried for 1 hr. at 50 °C. Later, 200 µl of lysis buffer containing 100 mM Tris at pH 8.5, 1 mg/ml of proteinase K and 0.05% of Tween 20 was added to the homogenate, vortexed and incubated at 60 °C in a water bath (Polystat – Cole Parmer Inc.) for 16 hrs. Post incubation, proteinase K was inactivated at 95 °C for 15 min. The lysate was cooled to room temperature and DNA was isolated by phenol chloroform extraction. DNeasy Kit (Cat No: 69504, Qiagen Inc. Netherlands) was used in samples where the tissue content was low.

### PCR and Detection of Mutations within the *rpoB* Gene

PCR was performed as described earlier^22^. Briefly, a 20 μl PCR reaction mix was prepared using 10 μl of Hot Start PCR Master Mix (Qiagen Inc. Netherlands), 2 μl of Q solution (Qiagen Inc. Netherlands), ≈2 μg of DNA and forward & reverse primers for *rpoB* gene, each at 0.25 μM concentration. The mixture was cycled 37 times on a thermal cycler at 94 °C for 1 min, 60 °C for 1 min and 70 °C for 1 min which was preceded by initial denaturation at 95 °C for 15 min and terminated by a final extension at 72 °C for 10 min. A 3–5 μl of the amplified PCR product was electrophoresed on 2% Agarose gel for detection of 276 bp *rpoB* amplicons. The presence of mutations within the amplified product was confirmed by DNA sequencing through a commercial agency – Scigenom Pvt. Ltd. at Cochin in Kerala, India. Sequence data was analyzed using MEGA Version-7 (Molecular Evolutionary Genetics Analysis) and Sequencher V. 5.4.6 The residue positions and mutations were numbered based on the reference strain (BR4923) of *M*. *leprae* with Genbank accession number: NC_002677.1.

### Comparative Modelling of RNAP of *M*. *leprae*

Comparative models for RNAP holoenzyme of *M*. *leprae* were generated using templates from *M*. *tuberculosis*^1^ (PDB ID:5UH5 (96% identity, 3.8 Å resolution), which contain RNAP and nucleic acid scaffold with DNA and three nucleotides of RNA complementary to the template DNA strand and PDB ID: 5UHC (96% identity, 4.0 Å resolution) containing all the elements similar to 5UH5 and rifampin). Molecular modelling was performed using Modeller 9.17^42^. We modelled all the subunits of RNAP of *M*. *leprae* (Fig. 1) and assembled them together with the synthetic nucleic acid scaffold containing a strand of non-template DNA, template DNA and a 3 nt stretch of RNA with and without rifampin using UCSF Chimera^43^. The modelled structures exhibited a root mean square deviation (RMSD) of 0.304 Å with PDB ID: 5UH5 (Crystal structure of *M*. *tuberculosis* transcription initiation complex containing 3 nt of RNA) and 0.203 Å with PDB ID: 5UHC (Crystal structure of *M*. *tuberculosis* transcription initiation complex containing 3 nt RNA in complex with rifampin).

The mutant models were generated using ANDANTE^35^, a program that uses χ angle conservation rules for the sidechains surpassing the need to screen a large rotamer library for proper orientation of side chains in comparative modelling. Fold assessments were performed using NDOPE^44^ and GA341^45^ methods. Additionally, the models were analyzed for quality using Rampage^46^, and Molprobity^47^. Interatomic interactions were analyzed using *in-house* developed tools, Arpeggio^48^ and Intermezzo (Bernardo O.M and Blundell T.L., unpublished). The models were built with 96% identity and 98% coverage. Assessment on Rampage indicated 96.6% in the favored regions and 3.1% in the allowed regions.

### Mutation Analysis

Structural and thermodynamic changes that occur due to point mutations in RNAP were analyzed using in-house developed tools namely mCSM^15^ and SDM^14^. Mutation cutoff scanning matrix (mCSM) relies on graph-based signatures. The signatures encode interatomic distance patterns that define the residue environment. These together with pharmacophore characteristics are used to train predictive models. This approach helped understanding the impact of mutations on protein stability (mCSM)^15^, protein-ligand interactions (mCSM-lig)^13^, protein-protein interfaces (mCSM-ppi)^15^ and protein-nucleic interactions (mCSM-NA)^49^. We also used other *in-house* tools such as Site Directed Mutator (SDM2)^14^, which predicts thermodynamic changes upon mutations using structural environment-dependent amino acid substitution tables and DUET^16^, which is a combination of mCSM and SDM.

### Normal Mode Analysis

Normal mode analysis (NMA) was performed to determine the covariance of alpha carbon atoms in various normal modes in the wild-type and mutant RNAP models. The impact of point mutations on protein stability and dynamics was analyzed using Elastic Network Contact Model (ENCoM)^28^, which uses a coarse-grained NMA to improve conformational space sampling. It accounts for the nature of the amino acids and introduces a pairwise atom type term proportional to the surface area that is in contact with the heavy atoms in the potential. It predicts conformational changes and remodels the protein in various flexible conformations using Modeller 9.17^42^ while considering each conformational state as a template. This method allows for proper placement of bond angles and spatial constraints for each model in a conformational ensemble. Normal modes were generated for wild-type and mutant models, and an RMSD (root mean square deviation) grid was built from the eigenvectors where the backbone RMSD fluctuated within a threshold amplitude of 2 Å. The 1129 (N) amino acids corresponding to the β subunit of RNAP were used to produce a matrix of 3 N x 3 N. Various interaction energies of the RNAP complex were determined using FoldX4^29^ with the R statistical package. R scripts from ENCoM^50^ were used to calculate changes in RMSD in different normal modes.

## Electronic supplementary material

Supplementary Material 1
