# Publisher Correction: Structural Implications of Mutations Conferring Rifampin Resistance in *Mycobacterium leprae*

**DOI:** 10.1038/s41598-018-26451-z

**Published:** 2018-05-23

**Authors:** Sundeep Chaitanya Vedithi, Sony Malhotra, Madhusmita Das, Sheela Daniel, Nanda Kishore, Anuja George, Shantha Arumugam, Lakshmi Rajan, Mannam Ebenezer, David B. Ascher, Eddy Arnold, Tom L. Blundell

**Affiliations:** 1 Department of Biochemistry, University of Cambridge, Tennis Court Rd., Cambridge, CB2 1GA UK; 2 Molecular Biology and Immunology Division, Schieffelin Institute of Health Research & Leprosy Center (SIH R & LC), Karigiri, Vellore, Tamil Nadu 632106 India; 3 Department of Dermatology, Father Muller Medical College & Hospital, Mangalore, Karnataka 575 002 India; 4 Department of Dermatology, Trivandrum Medical College, Trivandrum, Kerala 695011 India; 5 Department of Biochemistry and Molecular Biology, Bio21 Institute, University of Melbourne, Parkville, VIC 3052 Australia; 6 Center for Advanced Biotechnology and Medicine (CABM), and Rutgers University Department of Chemistry and Chemical Biology, 679 Hoes Lane, Piscataway, NJ 08854 USA

Correction to: *Scientific Reports* 10.1038/s41598-018-23423-1, published online 22 March 2018

This Article contains typographical errors.

In the Results section under subheading ‘Rifampin Binding in M. *leprae* RNAP’,

“As the activity of rifampin relies on its ability to induce a steric clash with the 5′-ribonucleotide, mutations that influence its orientation in the binding pocket might lead to reduction in these steric clashes resulting inseset_name sele rifampin resistance.”

should read:

“As the activity of rifampin relies on its ability to induce a steric clash with the 5′-ribonucleotide, mutations that influence its orientation in the binding pocket might lead to reduction in these steric clashes resulting in rifampin resistance.”

In the Discussion section,

“Lepromatous leprosy (LL) is characterized by high bacillary loads and low *M*. *leprae* specific cell-mediated immune responses^32^ in the host, increasing likelihood of the presence of bacterial persisters post-treatment which may adopt to rifampin by inducing point mutations with the RRDR.”

should read:

“Lepromatous leprosy (LL) is characterized by high bacillary loads and low *M*. *leprae* specific cell-mediated immune responses^32^ in the host, increasing likelihood of the presence of bacterial persisters post-treatment which may adapt to rifampin by inducing point mutations within the RRDR.”

