# Supplementary material for: Structural Implications of Mutations Conferring Rifampin Resistance in Mycobacterium leprae
Source: Sci Rep. 2018 Mar 22;8:5016. doi: 10.1038/s41598-018-23423-1 (PMC5864748; doi:10.1038/s41598-018-23423-1)
Supplement: Supplementary file 1 — Supplementary Material 1 [file 41598_2018_23423_MOESM1_ESM.pdf]

Title: Structural Implications of Mutations Conferring Rifampin Resistance in *Mycobacterium leprae.*

Running Title: *Vedithi S.C et al., RpoB Mutations –M. leprae 2017*

Authors: Sundeep Chaitanya Vedithi[1,2,*], Sony Malhotra[1], Madhusmita Das[2], Sheela Daniel[2], Nanda Kishore[3], Anuja George[4], Shantha Arumugam[2], Lakshmi Rajan[2], Mannam Ebenezer[2], David B Ascher[5], Eddy Arnold[6] & Tom L Blundell[1,*]

Affiliations:

1. Department of Biochemistry, University of Cambridge, Tennis Court Rd., CB2 1GA, UK
2. Molecular Biology and Immunology Division, Schieffelin Institute of Health Research & Leprosy Center (SIH R & LC), Karigiri, Vellore, Tamil Nadu – 632106, India.
3. Department of Dermatology, Father Muller Medical College & Hospital, Mangalore, Karnataka - 575 002, India
4. Department of Dermatology, Trivandrum Medical College, Trivandrum, Kerala – 695011, India
5. Department of Biochemistry and Molecular Biology, Bio21 Institute, University of Melbourne, Parkville, VIC 3052, Australia.
6. Center for Advanced Biotechnology and Medicine (CABM), and Rutgers University Department of Chemistry and Chemical Biology, 679 Hoes Lane, Piscataway, NJ 08854, USA

*** Corresponding Authors:**

Professor Sir Tom Blundell FRS
Director of Research and
Professor Emeritus of Biochemistry

Dr Sundeep Chaitanya Vedithi
Department of Biochemistry
Sanger Building
University of Cambridge
80 Tennis Ct. Rd., CB2 1GA, UK

Email: tom@cryst.bioc.cam.ac.uk; tlb20@cam.ac.uk;
scv26@cam.ac.uk
Ph: +44 1223 766033

***Mycobacterium leprae rpoB* gene Sequence:**

>NC_002677.1:c2276805-2273269 Mycobacterium leprae TN chromosome, complete genome
GTGCTGGAAGGATGCATCTTGCCAGATTTCGGCCAGAGCAAGACAGACGTTAGTCCTAGCCAGAGTCGCC
CCCAAAGTTCGCCCAACAACTCCGTGCCCGGCGCGCCCAACCGAATTTCATTTGCCAAGCTCCGCGAACC
GCTTGAGGTTCCGGGGCTACTTGATGTGCAGACTGATTCATTTGAGTGGTTGATCGGATCGCCGTGCTGG
CGTGCAGCGGCCGCAAGCCGCGGCGATCTCAAGCCGGTGGGTGGTCTCGAAGAGGTGCTCTACGAGCTGT
CGCCGATCGAGGATTTCTCCGGCTCAATGTCATTGTCTTTCTCCGATCCCCGTTTTGACGAAGTCAAGGC
GCCCGTCGAAGAGTGCAAAGACAAGGACATGACGTACGCGGCCCCGCTGTTCGTCACGGCCGAGTTCATC
AACAACAACACCGGGGAGATCAAGAGCCAGACGGTGTTTATGGGCGACTTCCCTATGATGACTGAGAAGG
GAACCTTCATCATCAACGGGACCGAGCGTGTCGTCGTTAGCCAGCTGGTGCGCTCCCCTGGAGTATACTT
CGACGAGACGATCGACAAGTCCACAGAAAAGACGCTGCATAGTGTCAAGGTGATTCCCAGCCGCGGTGCC
TGGTTGGAATTCGATGTCGATAAACGCGACACCGTCGGTGTCCGCATTGACCGGAAGCGCCGGCAACCCG
TCACGGTGCTTCTCAAAGCGCTAGGTTGGACCAGTGAGCAGATCACCGAGCGTTTCGGTTTCTCCGAGAT
CATGCGCTCGACGCTGGAGAAGGACAACACAGTTGGCACCGACGAGGCGCTGCTAGACATCTATCGTAAG
TTGCGCCCAGGTGAGCCGCCGACTAAGGAGTCCGCGCAGACGCTGTTGGAGAACCTGTTCTTCAAGGAGA
AACGCTACGACCTGGCCAGGGTTGGTCGTTACAAGGTCAACAAGAAGCTCGGGTTGCACGCCGGTGAGTT
GATCACGTCGTCCACGCTGACCGAAGAGGATGTCGTCGCCACCATAGAGTACCTGGTTCGTCTGCATGAG
GGTCAGTCGACAATGACTGTCCCAGGTGGGGTAGAAGTGCCAGTGGAAACTGACGATATCGACCACTTCG
GCAACCGCCGGCTGCGCACGGTCGGCGAATTGATCCAGAACCAGATCCGGGTCGGTATGTCGCGGATGGA
GCGGGTGGTCCGGGAGCGGATGACCACCCAGGACGTCGAGGCGATCACGCCGCAGACGCTGATCAATATC
CGTCCGGTGGTCGCCGCTATCAAGGAATTCTTCGGCACCAGCCAGCTGTCGCAGTTCATGGATCAGAACA
ACCCTCTGTCGGGCCTGACCCACAAGCGCCGGCTGTCGGCGCTGGGCCCGGGTGGTTTGTCGCGTGAGCG
TGCCGGGCTAGAGGTCCGTGACGTGCACCCTTCGCACTACGGCCGGATGTGCCCGATCGAGACTCCGGAG
GGCCCGAACATAGGTCTGATCGGTTCATTGTCGGTGTACGCGCGGGTCAACCCCTTCGGGTTCATCGAAA
CACCGTACCGCAAAGTGGTTGACGGTGTGGTCAGCGACGAGATCGAATACTTGACCGCTGACGAGGAAGA
CCGCCATGTCGTGGCGCAGGCCAACTCGCCGATCGACGAGGCCGGCCGCTTCCTCGAGCCGCGCGTGTTG
GTGCGCCGCAAGGCGGGCGAGGTGGAGTACGTGGCCTCGTCCGAGGTGGATTACATGGATGTCTCGCCAC
GCCAGATGGTGTCGGTGGCCACAGCGATGATTCCGTTCCTTGAGCACGACGACGCCAACCGTGCCCTGAT
GGGCGCTAACATGCAGCGCCAAGCGGTTCCGTTGGTGCGCAGCGAAGCACCGTTGGTGGGTACCGGTATG
GAGTTGCGCGCGGCCATCGACGCTGGCCACGTCGTCGTTGCGGAGAAGTCCGGGGTGATCGAGGAGGTTT
CCGCCGACTACATCACCGTGATGGCCGATGACGGCACCCGGCGGACTTATCGGATGCGTAAGTTCGCGCG
CTCCAACCACGGCACCTGCGCCAACCAGTCCCCGATCGTGGATGCGGGGGATCGGGTCGAGGCCGGCCAA
GTGATTGCTGACGGTCCGTGCACTGAGAACGGCGAGATGGCGTTGGGCAAGAACTTGCTGGTGGCGATCA
TGCCGTGGGAGGGTCACAACTACGAGGATGCGATCATCCTGTCTAACCGACTGGTCGAAGAGGACGTGCT
TACTTCGATTCACATTGAGGAGCATGAGATCGACGCCCGTGACACCAAGCTGGGTGCTGAGGAGATCACC
CGGGACATTCCCAACGTCTCCGATGAGGTGCTAGCCGACTTGGACGAGCGGGGCATCGTGCGGATTGGCG
CGGAGGTTCGTGACGGTGATATCCTGGTTGGCAAGGTCACCCCGAAGGGGGAAACTGAGCTGACACCGGA
AGAGCGGTTGCTGCGGGCGATCTTCGGCGAAAAGGCCCGCGAGGTCCGTGACACGTCGCTGAAGGTGCCA
CACGGCGAATCCGGCAAGGTGATCGGCATTCGGGTGTTCTCCCATGAGGATGACGACGAGCTGCCCGCCG
GCGTCAACGAGCTGGTCCGTGTCTACGTAGCCCAGAAGCGCAAGATCTCTGACGGTGACAAGCTGGCTGG
GCGGCACGGCAACAAGGGCGTGATCGGCAAGATCCTGCCTGCCGAGGATATGCCGTTTCTGCCAGACGGC
ACCCCGGTGGACATCATCCTCAACACTCACGGGGTGCCGCGGCGGATGAACGTCGGTCAGATCTTGGAAA
CCCACCTTGGGTGGGTAGCCAAGTCCGGCTGGAAGATCGACGTGGCCGGCGGTATACCGGATTGGGCGGT
CAACTTGCCTGAGGAGTTGTTGCACGCTGCGCCCAACCAGATCGTGTCGACCCCGGTGTTCGACGGCGCC
AAGGAAGAGGAACTACAGGGCCTGTTGTCCTCCACGTTGCCCAACCGCGACGGCGATGTGATGGTGGGCG
GCGACGGCAAGGCGGTGCTCTTCGATGGGCGCAGCGGTGAGCCGTTCCCTTATCCGGTGACGGTTGGCTA
CATGTACATCATGAAGCTGCACCACTTGGTGGACGACAAGATCCACGCCCGCTCCACCGGCCCGTACTCG
ATGATTACCCAGCAGCCGTTGGGTGGTAAGGCACAGTTCGGTGGCCAGCGATTCGGTGAGATGGAGTGCT
GGGCCATGCAGGCCTACGGTGCGGCCTACACGCTGCAGGAGCTGTTGACCATCAAGTCCGACGACACCGT
CGGTCGGGTCAAGGTTTACGAGGCTATCGTTAAGGGTGAGAACATCCCCGAGCCGGGCATCCCCGAGTCG
TTCAAGGTGCTGCTCAAGGAGTTACAGTCGCTGTGTCTCAACGTCGAGGTGCTGTCGTCCGACGGTGCGG
CGATCGAGTTGCGCGAAGGTGAGGATGAGGACCTCGAGCGGGCTGCGGCCAACCTCGGTATCAACTTGTC
CCGCAACGAATCGGCGTCCATAGAAGATCTGGCTTAG

RpoB Wt:(Highlighted in the gene above)

GTCGAGGCGATCACGCCGCAGACGCTGATCAATATCCGTCCGGTGGTCGCCGCTATCAAGGAATTCTTCGGCACC
AGCCAGCTGTCGCAGTTCATGGATCAGAACAACCCTCTGTCGGGCCTGACCCACAAGCGCCGGCTGTCGGCGCTG
GGCCCGGGTGGTTTGTCGCGTGAGCGTGCCGGGCTAGAGGTCCGTGACGTGCACCCTTCGCACTACGGCCGGATG
TGCCCGATCGAGACTCCGGAGGGCCCGAACATAGGTCTGATCGGTTCATTGTC

RpoB Wt. SEQUENCE ALIGNMENT:

```
Query  1       CGACAATGAACCGATCAGACCTATGTTCGGGCCCTCCGGAGTCTCGATCGGGCACATCCG  60
               ||||||||||||||||||||||||||||||||||||||||||||||||||||||||||||
Sbjct  276353  CGACAATGAACCGATCAGACCTATGTTCGGGCCCTCCGGAGTCTCGATCGGGCACATCCG  276412

Query  61      GCCGTAGTGCGAAGGGTGCACGTCACGGACCTCTAGCCCGGCACGCTCACGCGACAAACC  120
               ||||||||||||||||||||||||||||||||||||||||||||||||||||||||||||
Sbjct  276413  GCCGTAGTGCGAAGGGTGCACGTCACGGACCTCTAGCCCGGCACGCTCACGCGACAAACC  276472

Query  121     ACCCGGGCCCAGCGCCGACAGCCGGCGCTTGTGGGTCAGGCCCGACAGAGGGTTGTTCTG  180
               ||||||||||||||||||||||||||||||||||||||||||||||||||||||||||||
Sbjct  276473  ACCCGGGCCCAGCGCCGACAGCCGGCGCTTGTGGGTCAGGCCCGACAGAGGGTTGTTCTG  276532

Query  181     ATCCATGAACTGCGACAGCTGGCTGGTGCCGAAGAATTCCTTGATAGCGGCGACCACCGG  240
               ||||||||||||||||||||||||||||||||||||||||||||||||||||||||||||
Sbjct  276533  ATCCATGAACTGCGACAGCTGGCTGGTGCCGAAGAATTCCTTGATAGCGGCGACCACCGG  276592

Query  241     ACGGATATTGATCAGCGTCTGCGGCGTGATCGCCTCGAC  279
               |||||||||||||||||||||||||||||||||||||||
Sbjct  276593  ACGGATATTGATCAGCGTCTGCGGCGTGATCGCCTCGAC  276631
```

Translated Protein:

279 nucleotides, 93 amino acids, structure: sequence

```
  1 GTCGAGGCGATCACG CCGCAGACGCTGATC AATATCCGTCCGGTG GTCGCCGCTATCAAG
  1  V  E  A  I  T   P  Q  T  L  I   N  I  R  P  V   V  A  A  I  K
 61 GAATTCTTCGGCACC AGCCAGCTGTCGCAG TTCATGGATCAGAAC AACCCTCTGTCGGGC
 21  E  F  F  G  T   S  Q  L  S  Q   F  M  D  Q  N   N  P  L  S  G
121 CTGACCCACAAGCGC CGGCTGTCGGCGCTG GGCCCGGGTGGTTTG TCGCGTGAGCGTGCC
 41  L  T  H  K  R   R  L  S  A  L   G  P  G  G  L   S  R  E  R  A
181 GGGCTAGAGGTCCGT GACGTGCACCCTTCG CACTACGGCCGGATG TGCCCGATCGAGACT
 61  G  L  E  V  R   D  V  H  P  S   H  Y  G  R  M   C  P  I  E  T
241 CCGGAGGGCCCGAAC ATAGGTCTGATCGGT TCATTGTCG
 81  P  E  G  P  N   I  G  L  I  G   S  L  S
```

RpoB D441V: (Mutated Residue highlighted in Yellow)

```
>RpoB_26_forward
AGCTGTCGCAGTTCATGGTTCAGAACAACCCTCTGTCGGGCCTGACCCACAAGCGCCGGCTGTCGGCG
CTGGGCCCGGGTGGTTTGTCGCGTGAGCGTGCCGGGCTAGAGGTCCGTGACGTGCACCCTTCGCACTA
CGGCCGGATGTGCCCGATCGAGACTCCGGAGGGCCCGAACATAGGTCTGATCGGTTCATTGTCGA
```

RpoB D441V SEQUENCE ALIGNMENT:

```
Query  1       AGCTGTCGCAGTTCATGGTTCAGAACAACCCTCTGTCGGGCCTGACCCACAAGCGCCGGC  60
               |||||||||||||||||| |||||||||||||||||||||||||||||||||||||||||
Sbjct  276552  AGCTGTCGCAGTTCATGGATCAGAACAACCCTCTGTCGGGCCTGACCCACAAGCGCCGGC  276493

Query  61      TGTCGGCGCTGGGCCCGGGTGGTTTGTCGCGTGAGCGTGCCGGGCTAGAGGTCCGTGACG  120
               ||||||||||||||||||||||||||||||||||||||||||||||||||||||||||||
Sbjct  276492  TGTCGGCGCTGGGCCCGGGTGGTTTGTCGCGTGAGCGTGCCGGGCTAGAGGTCCGTGACG  276433

Query  121     TGCACCCTTCGCACTACGGCCGGATGTGCCCGATCGAGACTCCGGAGGGCCCGAACATAG  180
               ||||||||||||||||||||||||||||||||||||||||||||||||||||||||||||
Sbjct  276432  TGCACCCTTCGCACTACGGCCGGATGTGCCCGATCGAGACTCCGGAGGGCCCGAACATAG  276373

Query  181     GTCTGATCGGTTCATTGTCG  200
               ||||||||||||||||||||
Sbjct  276372  GTCTGATCGGTTCATTGTCG  276353
```

Translated Protein:

201 nucleotides, 66 amino acids, structure: AG sequence A

```
  1 CTGTCGCAGTTCATG GTTCAGAACAACCCT CTGTCGGGCCTGACC CACAAGCGCCGGCTG
  1  L  S  Q  F  M   V  Q  N  N  P   L  S  G  L  T   H  K  R  R  L
 61 TCGGCGCTGGGCCCG GGTGGTTTGTCGCGT GAGCGTGCCGGGCTA GAGGTCCGTGACGTG
 21  S  A  L  G  P   G  G  L  S  R   E  R  A  G  L   E  V  R  D  V
121 CACCCTTCGCACTAC GGCCGGATGTGCCCG ATCGAGACTCCGGAG GGCCCGAACATAGGT
 41  H  P  S  H  Y   G  R  M  C  P   I  E  T  P  E   G  P  N  I  G
181 CTGATCGGTTCATTG TCG
 61  L  I  G  S  L   S
```

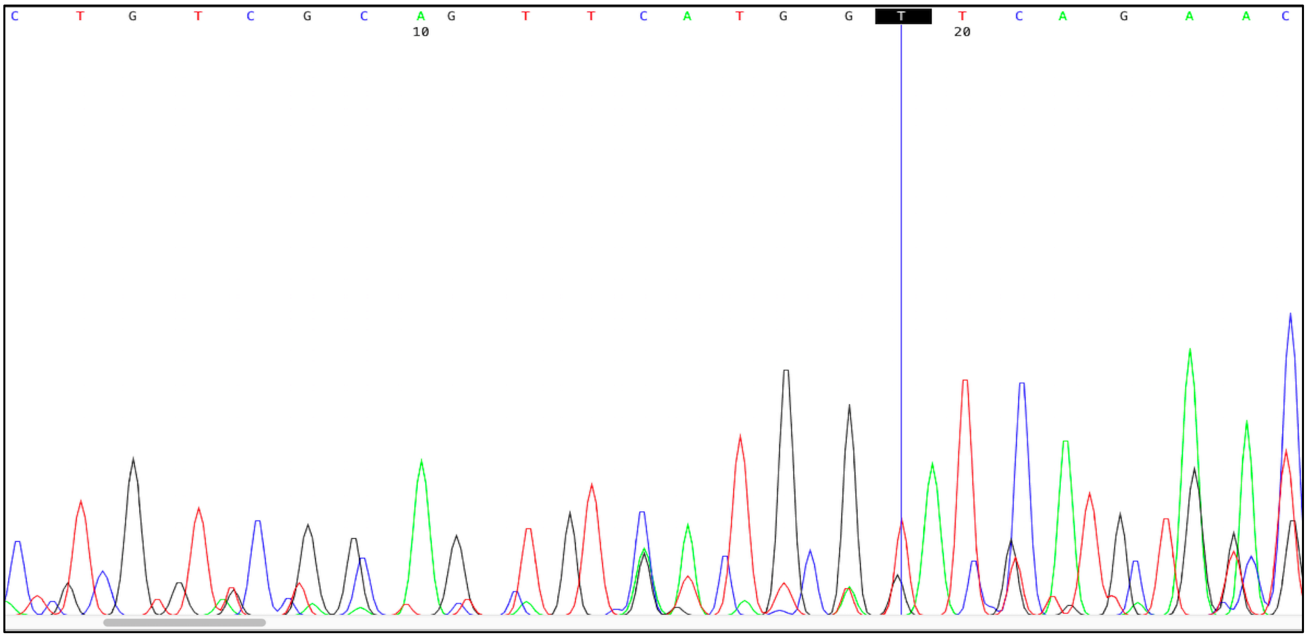

D441Y: (Mutated Residue highlighted in Yellow)

```
>RpoB_25
GATTCATGTATCAGAACAACCCTCTGTCGGGCCTGACCCACAAGCGCCGGCTGTCGGCGCTGGGCCCG
GGTGGTTTGTCGCGTGAGCGTGCCGGGCTAGAGGTCCGTGACGTGCACCCTTCGCACTACGGCCGGAT
GTGCCCGATCGAGACTCCGGAGGGCCCGAACATAGGTCTGATCGGTTCATTGTCGA
```

RpoB: D441Y: Sequence Alignment:

```
Query  3       TTCATGTATCAGAACAACCCTCTGTCGGGCCTGACCCACAAGCGCCGGCTGTCGGCGCTG  62
               |||||| |||||||||||||||||||||||||||||||||||||||||||||||||||||
Sbjct  276541  TTCATGGATCAGAACAACCCTCTGTCGGGCCTGACCCACAAGCGCCGGCTGTCGGCGCTG  276482

Query  63      GGCCCGGGTGGTTTGTCGCGTGAGCGTGCCGGGCTAGAGGTCCGTGACGTGCACCCTTCG  122
               ||||||||||||||||||||||||||||||||||||||||||||||||||||||||||||
Sbjct  276481  GGCCCGGGTGGTTTGTCGCGTGAGCGTGCCGGGCTAGAGGTCCGTGACGTGCACCCTTCG  276422

Query  123     CACTACGGCCGGATGTGCCCGATCGAGACTCCGGAGGGCCCGAACATAGGTCTGATCGGT  182
               ||||||||||||||||||||||||||||||||||||||||||||||||||||||||||||
Sbjct  276421  CACTACGGCCGGATGTGCCCGATCGAGACTCCGGAGGGCCCGAACATAGGTCTGATCGGT  276362

Query  183     TCATTGTCG  191
               |||||||||
Sbjct  276361  TCATTGTCG  276353
```

Translated Protein:

192 nucleotides, 63 amino acids, structure: GA sequence A

```
  1 TTCATGTATCAGAAC AACCCTCTGTCGGGC CTGACCCACAAGCGC CGGCTGTCGGCGCTG
  1  F  M  Y  Q  N   N  P  L  S  G   L  T  H  K  R   R  L  S  A  L
 61 GGCCCGGGTGGTTTG TCGCGTGAGCGTGCC GGGCTAGAGGTCCGT GACGTGCACCCTTCG
 21  G  P  G  G  L   S  R  E  R  A   G  L  E  V  R   D  V  H  P  S
121 CACTACGGCCGGATG TGCCCGATCGAGACT CCGGAGGGCCCGAAC ATAGGTCTGATCGGT
 41  H  Y  G  R  M   C  P  I  E  T   P  E  G  P  N   I  G  L  I  G
181 TCATTGTCG
 61  S  L  S
```

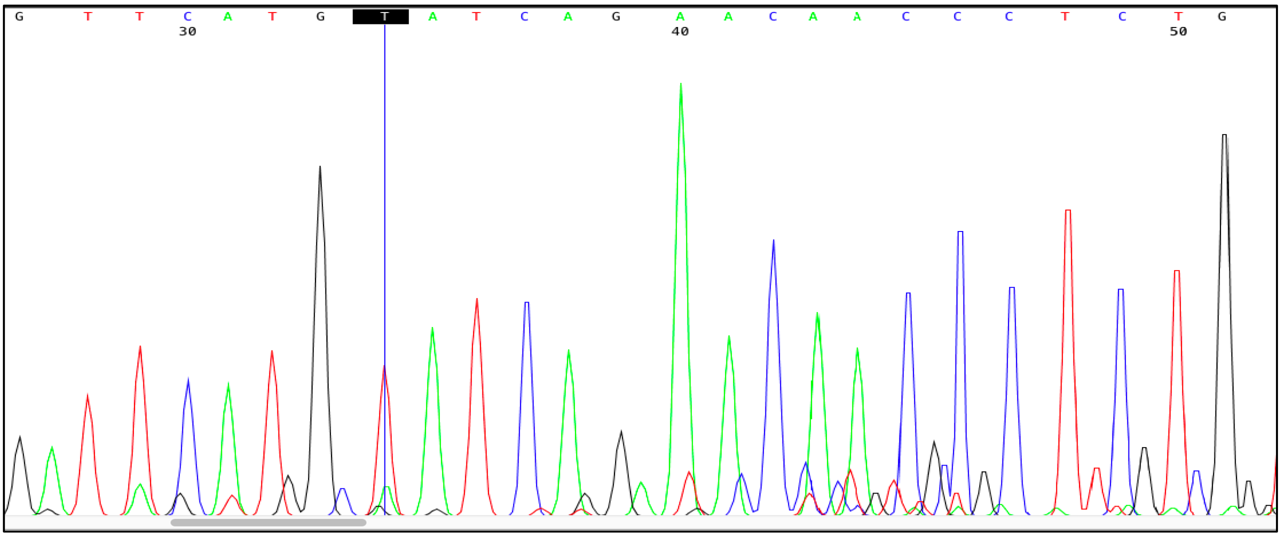

H476R: (Mutated Residue highlighted in Yellow)

```
GGGGCCCCGGTTAGTGCGAAGGGCGCACGTCACGGACCTCTAGCCCGGCACGCTCACGCGAC
AAACCACCCGGGCCCAGCGCCGACAGCCGGCGCTTGTGGGTCAGGCCCGACAGAGGGTTGTT
CTGATCCATGAACTGCGACAGCTGGCTGGTGCCGAAGAATTCCTTGATAGCGGCGACCACCG
GACGGATATTGATCAGCGTCTGCGGCGTGATCGCCTCGAC
```

```
Query  12      TAGTGCGAAGGGCGCACGTCACGGACCTCTAGCCCGGCACGCTCACGCGACAAACCACCC  71
               |||||||||||| |||||||||||||||||||||||||||||||||||||||||||||||
Sbjct  276417  TAGTGCGAAGGGTGCACGTCACGGACCTCTAGCCCGGCACGCTCACGCGACAAACCACCC  276476

Query  72      GGGCCCAGCGCCGACAGCCGGCGCTTGTGGGTCAGGCCCGACAGAGGGTTGTTCTGATCC  131
               ||||||||||||||||||||||||||||||||||||||||||||||||||||||||||||
Sbjct  276477  GGGCCCAGCGCCGACAGCCGGCGCTTGTGGGTCAGGCCCGACAGAGGGTTGTTCTGATCC  276536

Query  132     ATGAACTGCGACAGCTGGCTGGTGCCGAAGAATTCCTTGATAGCGGCGACCACCGGACGG  191
               ||||||||||||||||||||||||||||||||||||||||||||||||||||||||||||
Sbjct  276537  ATGAACTGCGACAGCTGGCTGGTGCCGAAGAATTCCTTGATAGCGGCGACCACCGGACGG  276596

Query  192     ATATTGATCAGCGTCTGCGGCGTGATCGCCTCGAC  226
               |||||||||||||||||||||||||||||||||||
Sbjct  276597  ATATTGATCAGCGTCTGCGGCGTGATCGCCTCGAC  276631
```

Translated Reverse frame 1

```
        226 nucleotides, 75 amino acids, structure:  sequence C
  1 GTCGAGGCGATCACG CCGCAGACGCTGATC AATATCCGTCCGGTG GTCGCCGCTATCAAG
  1  V  E  A  I  T   P  Q  T  L  I   N  I  R  P  V   V  A  A  I  K
 61 GAATTCTTCGGCACC AGCCAGCTGTCGCAG TTCATGGATCAGAAC AACCCTCTGTCGGGC
 21  E  F  F  G  T   S  Q  L  S  Q   F  M  D  Q  N   N  P  L  S  G
121 CTGACCCACAAGCGC CGGCTGTCGGCGCTG GGCCCGGGTGGTTTG TCGCGTGAGCGTGCC
 41  L  T  H  K  R   R  L  S  A  L   G  P  G  G  L   S  R  E  R  A
181 GGGCTAGAGGTCCGT GACGTGCGCCCTTCG CACTAACCGGGGCCC
 61  G  L  E  V  R   D  V  R  P  S   H  *  P  G  P
```

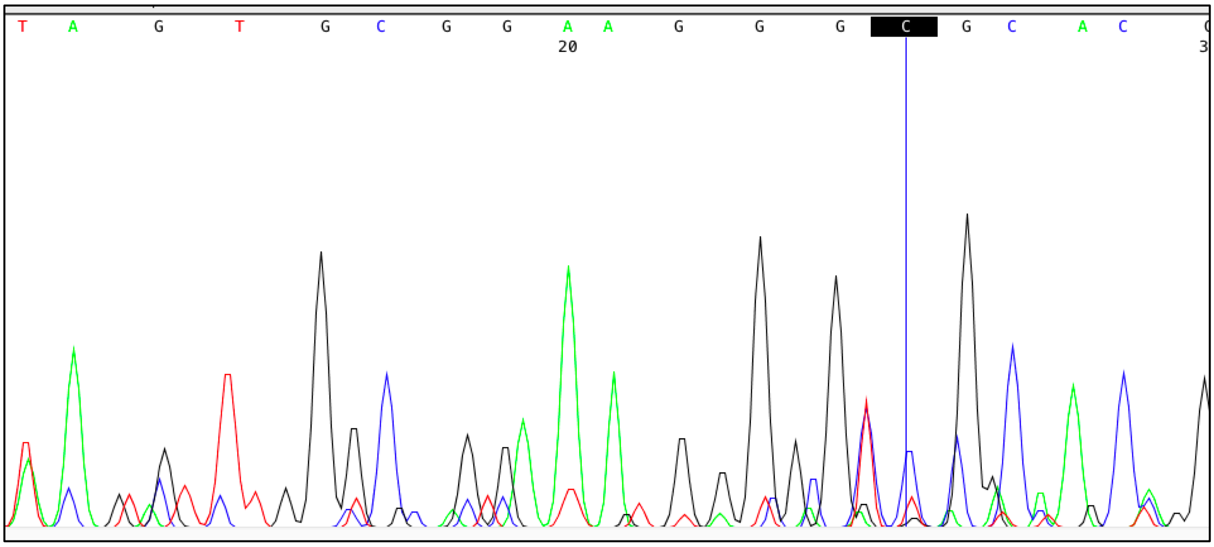

S437L: (Mutated Residue highlighted in Yellow)

CCGGCGCTTGTGGGTCAGGCCCGACAGAGGGTTGTTCTGATCCATGAACTGC**A**ACAGCTGGCTGGTGC
CGAAGAATTCCTTGATAGCGGCGACCACCGGACGGATATTGATCAGCGTCTGCGGCGTGATCGCCTCG
AC

```
Query  1       CCGGCGCTTGTGGGTCAGGCCCGACAGAGGGTTGTTCTGATCCATGAACTGCAACAGCTG  60
               |||||||||||||||||||||||||||||||||||||||||||||||||||| |||||||
Sbjct  276494  CCGGCGCTTGTGGGTCAGGCCCGACAGAGGGTTGTTCTGATCCATGAACTGCGACAGCTG
276553

Query  61      GCTGGTGCCGAAGAATTCCTTGATAGCGGCGACCACCGGACGGATATTGATCAGCGTCTG  120
               ||||||||||||||||||||||||||||||||||||||||||||||||||||||||||||
Sbjct  276554  GCTGGTGCCGAAGAATTCCTTGATAGCGGCGACCACCGGACGGATATTGATCAGCGTCTG
276613

Query  121     CGGCGTGATCGCCTCGAC  138
               ||||||||||||||||||
Sbjct  276614  CGGCGTGATCGCCTCGAC  276631
```

Translated Reverse frame 1

```
                  138 nucleotides, 46 amino acids, structure:  sequence
 1 GTCGAGGCGATCACG CCGCAGACGCTGATC AATATCCGTCCGGTG GTCGCCGCTATCAAG GAATTCTTCGGCACC
 1  V  E  A  I  T   P  Q  T  L  I   N  I  R  P  V   V  A  A  I  K   E  F  F  G  T
76 AGCCAGCTGTTGCAG TTCATGGATCAGAAC AACCCTCTGTCGGGC CTGACCCACAAGCGC CGG
26  S  Q  L  L  Q   F  M  D  Q  N   N  P  L  S  G   L  T  H  K  R   R
```

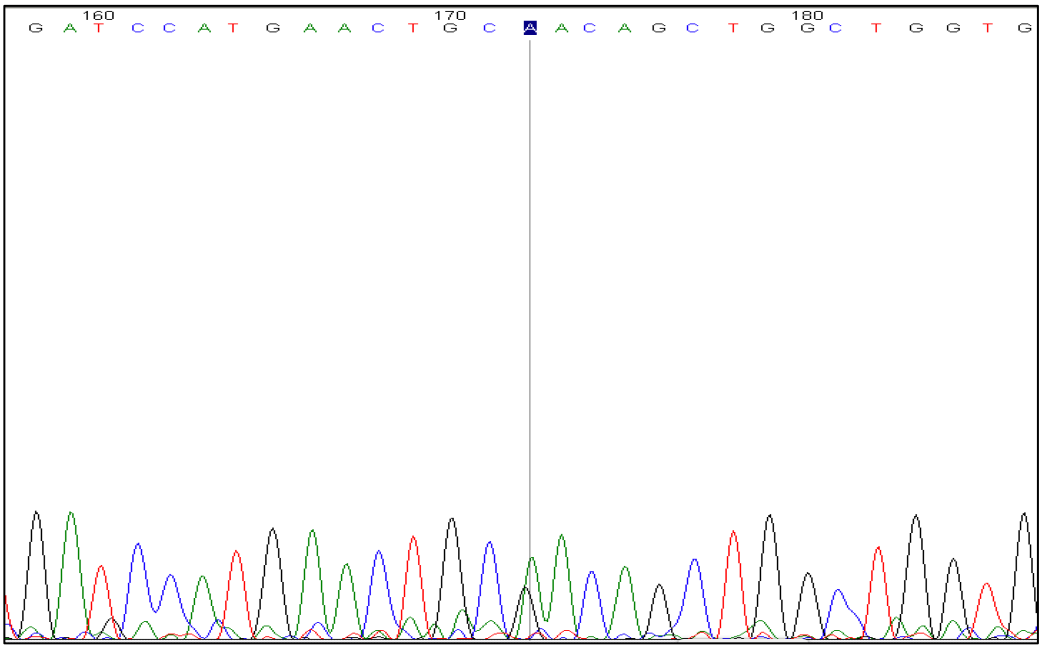

II: Multiple sequence alignment of the β-subunit of RNA Polymerase in Mycobacterial Species *M. abscessus, M. ulcerans, M. kansasii, M. leprae and M. tuberculosis.*

(The residue positions for mutations were highlighted in yellow)

```
CLUSTAL multiple sequence alignment by MUSCLE (3.8)

abscessus        -MAGQVVQYGRHRK------------------------RRNYARISEVLELPNLIEIQTKS
ulcerans         MLEGRILADFRQTD--ASLHQGRPQSSSNSSVPGAPNRVSFAKLREPLEVPGLLDVQTDS
kansasii         MLEGRILADFRQSKTDASPAQSRPQSSSNSSVPGAPNRVSFAKLREPLEVPGLLDVQTDS
leprae           MLEGCILPDFGQSKTDVSPSQSRPQSSPNNSVPGAPNRISFAKLREPLEVPGLLDVQTDS
tuberculosis     MLEGCILADSRQSKTAASPSPSRPQSSSNNSVPGAPNRVSFAKLREPLEVPGLLDVQTDS
                  : * ::    : .                       * .:*.: * **:*.*:::**.*

abscessus        YDWFLKE---------------------GLLEMFRDISPIEDFTGNLSLEFVDYRLGEPK
ulcerans         FEWLIGSQRWRESAAQRGD----ATPVGGLEEVLYELSPIEDFSGSMSLSFSDPRFDEVK
kansasii         FEWLIGSQRWRDSAIARGEAAGGINPIGGLEEVLDELSPIEDFSGSMSLSFSDPRFDEVK
leprae           FEWLIGSPCWRAAAAASRGD----LKPVGGLEEVLYELSPIEDFSGSMSLSFSDPRFDEVK
tuberculosis     FEWLIGSPRWRESAAERGD----VNPVGGLEEVLYELSPIEDFSGSMSLSFSDPRFDDVK
                 ::*:: .                     ** *:: ::******:*.:**.* * *:.: *

abscessus        YDLEESKNRDATYAAPLRVKVRLIIKETGEVKEQEVFMGDFPLMTDTGTFVINGAERVIV
ulcerans         APVDECKDKDMTYAAPLFVTAEFINNNTGEIKSQTVFMGGFPMMTEKGTFIINGTERVVV
kansasii         APVDECKDKDMTYAAPLFVTAEFINNNTGEIKSQTVFMGDFPMMTEKGTFIINGTERVVV
leprae           APVEECKDKDMTYAAPLFVTAEFINNNTGEIKSQTVFMGDFPMMTEKGTFIINGTERVVV
tuberculosis     APVDECKDKDMTYAAPLFVTAEFINNNTGEIKSQTVFMGDFPMMTEKGTFIINGTERVVV
                   ::*.*:.* ****** *.. :* ::***:*.* ****.**:**:.***:***:***:*

abscessus        SQLVRSPSVYFNEKIDKN-GRENYDATIIPNRGAWLEYETDAKDIVYVRIDRTRKLPLTV
ulcerans         SQLVRSPGVYFDETIDKSTDKLLHSVKVIPSRGAWLEFDVDKRDTVGVRIDRKRRQPVTV
kansasii         SQLVRSPGVYFDETIDKSTDKTLHSVKVIPSRGAWLEFDVDKRDTVGVRIDRKRRQPVTV
leprae           SQLVRSPGVYFDETIDKSTEKTLHSVKVIPSRGAWLEFDVDKRDTVGVRIDRKRRQPVTV
tuberculosis     SQLVRSPGVYFDETIDKSTDKTLHSVKVIPSRGAWLEFDVDKRDTVGVRIDRKRRQPVTV
                 *******.***:*.***.  .   :...:**.******::.* .* * *****.*. *:**

abscessus        LLRALGFSSDQEIIDLLGDSEYLRNTLEKDSTENTEQALLEIYERLRPGEPPTVENAKSL
ulcerans         LLKALGWSNEQ-IHERFGFSEIMMGTLEKDNTAGTDEALLDIYRKLRPGEPPTKESAQTL
kansasii         LLKALGWTSER-ITERFGFSEIMMSTLEKDNTAGTDEALLDIYRKLRPGEPPTKESAQTL
leprae           LLKALGWTSEQ-ITERFGFSEIMRSTLEKDNTVGTDEALLDIYRKLRPGEPPTKESAQTL
tuberculosis     LLKALGWTSEQ-IVERFGFSEIMRSTLEKDNTVGTDEALLDIYRKLRPGEPPTKESAQTL
                 **.***::.:. * : :* ** : .*****.* .*::***:** .******** *.*::*

abscessus        LYSRFFDPKRYDLASVGRYKTNKKLHLKHRLFNQKLAEPIVDSETGEIVAEEGTVLDRRK
ulcerans         LENLFFKEKRYDLARVGRYKVNKKLGL-------NAGQPITSS-----------------
kansasii         LENLFFKEKRYDLARVGRYKVNKKLGL-------NTNHPITTT-----------------
leprae           LENLFFKEKRYDLARVGRYKVNKKLGL-------HAGELITSS-----------------
tuberculosis     LENLFFKEKRYDLARVGRYKVNKKLGL-------HVGEPITSS-----------------
                 * . **. ****** *****.**** *       :    *. :

abscessus        LDEIMNVLESNANSEVFELEGSVIDEPVEIQSIKVYVPNDEEERTTTVIGNALPDSEVKC
ulcerans         ---------------------TLTEEDV-VATIEYLVRLHEGQTAMTAPGGVEVPVE---
kansasii         ---------------------TLTEEDV-VATIEYLVRLHEGQATMTVPGGVEVPVE---
leprae           ---------------------TLTEEDV-VATIEYLVRLHEGQSTMTVPGGVEVPVE---
tuberculosis     ---------------------TLTEEDV-VATIEYLVRLHEGQTTMTVPGGVEVPVE---
                                      :: :* * : :*:  *   * : : *. *..    *

abscessus        ITPADIIASMSYFFNLLNGIGYTDDIDHLGNRRLRSVGELLQNQFRIGLSRMERVVRERM
ulcerans         ----------------------TDDIDHFGNRRLRTVGELIQNQIRVGMSRMERVVRERM
kansasii         ----------------------TDDIDHFGNRRLRTVGELIQNQIRVGMSRMERVVRERM
leprae           ----------------------TDDIDHFGNRRLRTVGELIQNQIRVGMSRMERVVRERM
tuberculosis     ----------------------TDDIDHFGNRRLRTVGELIQNQIRVGMSRMERVVRERM
                                       ******:******:****:***:*:*:**********

                                                 437 441
abscessus        SIQDTDSITPQQLINIRPVIASIKEFFGSSQLSQFMDQANPLAELTHKRRLSALGPGGLT
ulcerans         TTQDVEAITPQTLINIRPVVAAIKEFFGTSQLSQFMDQNNPLSGLTHKRRLSALGPGGLS
kansasii         TTQDVEAITPQTLINIRPVVAAIKEFFGTSQLSQFMDQNNPLSGLTHKRRLSALGPGGLS
leprae           TTQDVEAITPQTLINIRPVVAAIKEFFGTSQLSQFMDQNNPLSGLTHKRRLSALGPGGLS
tuberculosis     TTQDVEAITPQTLINIRPVVAAIKEFFGTSQLSQFMDQNNPLSGLTHKRRLSALGPGGLS
                 : **.::**** *******:*:******:********* ***: **************:

                           476
abscessus        RERAQMEVRDVHYSHYGRMCPIETPEGPNIGLINSLSSYARVNEFGFIETPYRKVDIDTN
ulcerans         RERAGLEVRDVHPSHYGRMCPIETPEGPNIGLIGSLSVYARVNPFGFIETPYRKV-VD-G
kansasii         RERAGLEVRDVHPSHYGRMCPIETPEGPNIGLIGSLSVYARVNPFGFIETPYRKV-ID-G
leprae           RERAGLEVRDVHPSHYGRMCPIETPEGPNIGLIGSLSVYARVNPFGFIETPYRKV-VD-G
tuberculosis     RERAGLEVRDVHPSHYGRMCPIETPEGPNIGLIGSLSVYARVNPFGFIETPYRKV-VD-G
                 **** :****** *******************.*** ***** *********** :* .
```

```
abscessus       SITDQIDYLTADEEDSYVVAQANSRLDENGRFLDDEVVCRFR-GNNTVMAKEKMDYMDVS
ulcerans        VVSDEIHYLTADEEDRHVVAQANSPIDAQGRFVEPRVLVRRKAGEVEYVPSSEVDYMDVS
kansasii        LVTDEIHYLTADEEDRHVVAQANSPIDAEGRFVEPRVLVRRKAGEVEYVASSEVDYMDVS
leprae          VVSDEIEYLTADEEDRHVVAQANSPIDEAGRFLEPRVLVRRKAGEVEYVASSEVDYMDVS
tuberculosis    VVSDEIVYLTADEEDRHVVAQANSPIDADGRFVEPRVLVRRKAGEVEYVPSSEVDYMDVS
                 ::*:* ******** :******* :*  ***::  *: * . *:   :...::******

abscessus       PKQVVSAATACIPFLENDDSNRALMGANMQRQAVPLMNPEAPFVGTGMEHVAARDSGAAI
ulcerans        PRQMVSVATAMIPFLEHDDANRALMGANMQRQAVPLVRSEAPLVGTGMELRAAIDAGDVV
kansasii        PRQMVSVATAMIPFLEHDDANRALMGANMQRQAVPLVRSEAPLVGTGMELRAAIDAGDVV
leprae          PRQMVSVATAMIPFLEHDDANRALMGANMQRQAVPLVRSEAPLVGTGMELRAAIDAGHVV
tuberculosis    PRQMVSVATAMIPFLEHDDANRALMGANMQRQAVPLVRSEAPLVGTGMELRAAIDAGDVV
                *.*:**.*** *****:**:***************:..***:******  ** *:* .:

abscessus       TAKHRGRVEHVESNEILVRRLVEENGTEHEGELDRYPLAKFKRSNSGTCYNQRPIVSVGD
ulcerans        VADKAGVIEEVSADYITV---MADDGTRHT-----YRMRKFARSNHGTCANQSPIVDAGE
kansasii        VAEKSGVIEEVSADYITV---MADDGTRHT-----YRMRKFARSNHGTCANQSPIVDAGE
leprae          VAEKSGVIEEVSADYITV---MADDGTRRT-----YRMRKFARSNHGTCANQSPIVDAGD
tuberculosis    VAEESGVIEEVSADYITV---MHDNGTRRT-----YRMRKFARSNHGTCANQCPIVDAGD
                .*.  * :* *.:: * *   : ::** .      * : ** *** *** ** ***..*:

abscessus       VVEYNEILADGPSMELGEMALGRNVVVGFMTWDGYNYEDAVIMSERLVKDDVYTSIHIEE
ulcerans        RVEAGQVIADGPCTQNGEMALGKNLLVAIMPWEGHNYEDAIILSNRLVEEDVLTSIHIEE
kansasii        RVEAGQVIADGPCTQNGEMALGKNLLVAIMPWEGHNYEDAIILSNRLVEEDILTSIHIEE
leprae          RVEAGQVIADGPCTENGEMALGKNLLVAIMPWEGHNYEDAIILSNRLVEEDVLTSIHIEE
tuberculosis    RVEAGQVIADGPCTDDGEMALGKNLLVAIMPWEGHNYEDAIILSNRLVEEDVLTSIHIEE
                 ** .:::****. : ******.*::*.:*.*:*:*****:*:*:***::*: *******

abscessus       YESEARDTKLGPEEITRDIPNVSENALKNLDDRGIIYVGAEVKDGDILVGKVTPKGVTEL
ulcerans        HEIDARDTKLGAEEITRDIPNVSDEVLADLDERGIVRIGAEVRDGDILVGKVTPKGETEL
kansasii        HEIDARDTKLGAEEITRDIPNVSDEVLADLDERGIVRIGAEVRDGDILVGKVTPKGETEL
leprae          HEIDARDTKLGAEEITRDIPNVSDEVLADLDERGIVRIGAEVRDGDILVGKVTPKGETEL
tuberculosis    HEIDARDTKLGAEEITRDIPNISDEVLADLDERGIVRIGAEVRDGDILVGKVTPKGETEL
                :* :*******.********:*::.* :**:***: :****.************* ***

abscessus       TAEERLLHAIFGEKAREVRDTSLRVPHGAGGIVLDVKVFNREEGDDTLSPGVNQLVRVYI
ulcerans        TPEERLLRAIFGEKAREVRDTSLKVPHGESGKVIGIRVFSRED-DDELPAGVNELVRVYV
kansasii        TPEERLLRAIFGEKAREVRDTSLKVPHGESGKVIGIRVFSRED-DDELPAGVNELVRVYV
leprae          TPEERLLRAIFGEKAREVRDTSLKVPHGESGKVIGIRVFSHED-DDELPAGVNELVRVYV
tuberculosis    TPEERLLRAIFGEKAREVRDTSLKVPHGESGKVIGIRVFSRED-EDELPAGVNELVRVYV
                *.*****.**************.****  .* *:.:.**..*: :* *..***:*****:

abscessus       VQKRKIHVGDKMCGRHGNKGVISKIVPEEDMPYLPDGRPIDIMLNPLGVPSRMNIGQVLE
ulcerans        AQKRKISDGDKLAGRHGNKGVIGKILPAEDMPFLPDGTPVDIILNTHGVPRRMNIGQILE
kansasii        AQKRKISDGDKLAGRHGNKGVIGKILAQEDMPFLPDGTPVDIILNTHGVPRRMNIGQILE
leprae          AQKRKISDGDKLAGRHGNKGVIGKILPAEDMPFLPDGTPVDIILNTHGVPRRMNVGQILE
tuberculosis    AQKRKISDGDKLAGRHGNKGVIGKILPVEDMPFLADGTPVDIILNTHGVPRRMNIGQILE
                .*****  ***:.*********.**:. ****:*.** *:**:**. *** ***:**:**

abscessus       LHLGMAAKN-------LGIH------------------VASPVFDGANDDDVWSTIE---
ulcerans        THLGWVAKSGWNIDVANGVPEWAGKLPENLLSAQPDSIVSTPVFDGAQEAELQGLLSATL
kansasii        THLGWCAHSGWQI---AGSPDWAANLPEALRSAEPNQIVSTPVFDGAQEAELQGLLSSTL
leprae          THLGWVAKSGWKIDVAGGIPDWAVNLPEELLHAAPNQIVSTPVFDGAKEEELQGLLSSTL
tuberculosis    THLGWCAHSGWKVDAAKGVPDWAARLPDELLEAQPNAIVSTPVFDGAQEAELQGLLSCTL
                 ***  *:.        *                    *::******:: ::.. :.

abscessus       -----EAGMARDGKTVLYDGRTGEPFDNRISVGVMYMLKLAHMVDDKLHARSTGPYSLVT
ulcerans        PNRDGEVLVDGDGKAKLFDGRSGEPFPYPVTVGYMYIMKLHHLVDDKIHARSTGPYSMIT
kansasii        PNRDGDVLVDGDGKAVLFDGRSGEPFPYPVTVGYMYIMKLHHLVDDKIHARSTGPYSMIT
leprae          PNRDGDVMVGGDGKAVLFDGRSGEPFPYPVTVGYMYIMKLHHLVDDKIHARSTGPYSMIT
tuberculosis    PNRDGDVLVDADGKAMLFDGRSGEPFPYPVTVGYMYIMKLHHLVDDKIHARSTGPYSMIT
                     :. :  ***: *:***:****   ::** **::** *:****:*********::*

abscessus       QQPLGGKAQFGGQRFGEMEVWALEAYGAAYTLQEILTYKSDDTVGRVKTYESIVKGENIS
ulcerans        QQPLGGKAQFGGQRFGEMECWAMQAYGAAYTLQELLTIKSDDTVGRVKVYEAIVKGENIP
kansasii        QQPLGGKAQFGGQRFGEMECWAMQAYGAAYTLQELLTIKSDDTVGRVKVYEAIVKGENIP
leprae          QQPLGGKAQFGGQRFGEMECWAMQAYGAAYTLQELLTIKSDDTVGRVKVYEAIVKGENIP
tuberculosis    QQPLGGKAQFGGQRFGEMECWAMQAYGAAYTLQELLTIKSDDTVGRVKVYEAIVKGENIP
                ****************** **::**********:** **********.**:*******.

abscessus       RPSVPESFRVLMKELQSLGLDVKVMDEHDNEIEMADVDDED----ATERKVDLQQKDVPE
ulcerans        EPGIPESFKVLLKELQSLCLNVEVLSSDGAAIELREGEDEDLERAAANLGINLSRNESAS
```

```
kansasii          EPGIPESFKVLLKELQSLCLNVEVLSSDGAAIELREGEDEDLERAAANLGINLSRNESAS
leprae            EPGIPESFKVLLKELQSLCLNVEVLSSDGAAIELREGEDEDLERAAANLGINLSRNESAS
tuberculosis      EPGIPESFKVLLKELQSLCLNVEVLSSDGAAIELREGEDEDLERAAANLGINLSRNESAS
                   *.:****.**:****** *:*:*:.. .  **: : :***    *::  ::*..:: ..

abscessus         TQKETNE
ulcerans          VEDLA--
kansasii          VEDLA--
leprae            IEDLA--
tuberculosis      VEDLA--
                   :. :
```
